# Genomics‐assisted breeding for successful development of multiple‐stress‐tolerant, climate‐smart rice for southern and southeastern Asia

**DOI:** 10.1002/tpg2.20074

**Published:** 2021-01-12

**Authors:** Shailesh Yadav, Nitika Sandhu, Shalabh Dixit, Vikas Kumar Singh, Margaret Catolos, Ratna Rani Mazumder, Mohammad Akhlasur Rahman, Arvind Kumar

**Affiliations:** ^1^ Rice Breeding Platform International Rice Research Institute DAPO Box 7777 Manila Philippines; ^2^ Punjab Agricultural University Ludhiana Punjab India; ^3^ International Rice Research Institute, South Asia Hub ICRISAT Patancheru Hyderabad India; ^4^ Plant Breeding Division, Bangladesh Rice Research Institute (BRRI) Gazipur Bangladesh; ^5^ IRRI South Asia Regional Centre (ISARC) Varanasi Uttar Pradesh 221106 India

## Abstract

Rice (*Oryza sativa* L.) in rainfed marginal environments is prone to multiple abiotic and biotic stresses, which can occur in combination in a single cropping season and adversely affect rice growth and yield. The present study was undertaken to develop high‐yielding, climate‐resilient rice that can provide tolerance to multiple biotic and abiotic stresses. An assembled first‐crossing scheme was employed to transfer 15 quantitative trait loci (QTL) and genes—*qDTY_1.1_*, *qDTY_2.1_*, *qDTY_3.1_*, *qDTY_12.1_* (drought), *Sub1* (submergence), *Gm4* (gall midge), *Pi9*, *Pita2* (blast), *Bph3*, *Bph17* (brown plant hoppers), *Xa4*, *xa5*, *xa13*, *Xa21*, and *Xa23* (bacterial leaf blight)—from eight different parents using genomics‐assisted breeding. A funnel mating design was employed to assemble all the targeted QTL and genes into a high‐yielding breeding line IR 91648‐B‐1‐B‐3‐1. Gene‐based linked markers were used in each generation from intercrossing to the F_6_ generation for tracking the presence of desirable alleles of targeted QTL and genes. Single‐plant selections were performed from F_2_ onwards to select desirable recombinants possessing alleles of interest with suitable phenotypes. Phenotyping of 95 homozygous F_6_ lines carrying six to 10 QTL and genes was performed for nonstress, reproductive‐stage (RS) drought, blast, bacterial leaf blight (BLB), gall midge (GM), and for grain quality parameters such as chalkiness, amylose content (AC), gelatinization temperature (GT), and head rice recovery (HRR). Finally, 56 F_7_ homozygous lines were found promising for multiple‐location evaluation for grain yield (GY) and other traits. These multiple‐stress‐tolerant lines with the desired grain quality profiling can be targeted for varietal release in southern and southeastern Asia through national release systems.

## Core Ideas

- Genomics‐assisted breeding used to assemble multiple genes and QTL for high‐yielding breeding line.
- High‐yielding, climate‐resilient, superior quality rice lines with 6–10 genes and QTL for various stresses.
- New lines used as donor parent to introgress multiple genes and QTL in other genetic backgrounds.

**Abbreviations**
AC: amylose content
BLB: bacterial leaf blight
BPH: brown plant hopper
BRRI: Bangladesh Rice Research Institute
DS: dry season
DTF: days to flowering
GM: gall midge
GT: gelatinization temperature
GY: grain yield
*H* ^2^
: broad‐sense heritability
HRR: head rice recovery
IRRI: International Rice Research Institute
L/W: length‐to‐width
PH: plant height
QTL: quantitative trait loci
RS: reproductive‐stage
SES: standard evaluation system
SNP: single nucleotide polymorphism
WS: wet season
*Xoo*
: *Xanthomonas oryzae* pv. *oryzae*

## 1 INTRODUCTION

Food crop production and nutritional properties will be consistently and negatively affected by climate change. Crop plants are likely to face the threat of occurrence of multiple stresses. The effect of combined stresses on crop plants is more complex, unpredictable, and leads to higher losses in grain yield (GY) depending upon interactions between different abiotic, biotic, or abiotic–biotic stresses (Pandey, Ramegowda, & Senthil, 2015). Several reports claimed the effect of concurrent occurrence of abiotic stresses in a single crop season is most damaging to crop growth and productivity (Atkinson, Lilley, & Urwin, 2013; Mahalingam, 2015). For instance, the concurrent occurrence of drought and flood can destroy rice (*Oryza sativa* L.) production in many of the rainfed areas of southern and southeastern Asia (Rahman & Zhang, 2016). Rice crops face many abiotic and biotic stresses during different stages of development, and further increases in the number and intensity of various abiotic and biotic stresses are anticipated as a result of ongoing climate change. The impact of global temperature increase with each degree Celsius has been predicted to reduce the global rice yield by 3.2% (Zhao et al., 2017). Climate‐resilient rice varieties with enhanced tolerance to extreme climatic changes, such as drought, flooding or high temperature stresses, are essential to sustain and improve rice yields under multiple challenges of climate change.

With the advent of molecular‐marker technology, the International Rice Research Institute (IRRI) achieved a tremendous success in identifying stable GY QTL under drought, that is, *qDTY_1.1_*, *qDTY_2.1_*, *qDTY_2.2_*, *qDTY_3.1_*, *qDTY_3.2_*, *qDTY_12.1_* (Venuprasad et al., 2009; Vikram et al., 2011), and flood‐tolerant gene *Sub1* (Septiningsih et al., 2015; Xu & Mackill, 1996), which have already been used in various rice breeding programs (Dixit et al., 2017; Kumar et al., 2014; Sandhu et al., 2019). In recent years, the transfer of some of the drought QTL and flood‐tolerant gene *Sub1* has been used extensively through a marker‐assisted backcross approach and have successfully led to the development of the drought or flood‐tolerant version of some of the mega varieties such as ‘Swarna’, ‘IR64’, and ‘Sambha Mahsuri’. Some of these developed breeding lines are released as varieties in various countries of southern and southeastern Asia for cultivation (Kumar et al., 2014, 2018; Septiningsih et al., 2015).

**Core Ideas**

- Genomics‐assisted breeding used to assemble multiple genes and QTL for high‐yielding breeding line.
- High‐yielding, climate‐resilient, superior quality rice lines with 6–10 genes and QTL for various stresses.
- New lines used as donor parent to introgress multiple genes and QTL in other genetic backgrounds.

Among biotic stresses, rice is most vulnerable to incidence of bacterial leaf blight (BLB) caused by *Xanthomonas oryzae* pv. *Oryzae* (*Xoo*), which can cause partial grain filling as a result of limited photosynthetic activity (Ou, 1985), resulting in GY losses up to 50% under favorable conditions (Liu, Liu, Triplett, Leach, & Wang, 2014). Use of gene‐conferred resistant rice varieties provides an economical, effective, and environment‐friendly approach to protect the crop from this disease and minimize losses. So far, ∼43 BLB‐resistant (*R*) genes have been identified in rice diverse germplasm (Dilla‐Ermita et al., 2017) and 11 of them have been isolated, cloned, and fine mapped by using the recent techniques available in modern genetics and genomics (Tian et al., 2014). Using a gene‐pyramiding approach, various BLB resistance genes in combinations of either three or four genes were incorporated in modern high‐yielding rice varieties to achieve broader and more durable resistance (Dokku, Das, & Rao, 2013; Pradhan et al., 2015; Suh et al., 2013; Sundaram et al., 2008). Bacterial leaf blight gene combinations, such as *Xa4*, *xa5*, *xa13*, and *Xa21*, were found highly stable and confers resistance to most of the isolates of the pathogen (Shanti & Shenoy,2005; Dokku et al., 2013). One of the dominant BLB gene, *Xa23* used in the current introgression program, confers broader resistance in combination with other *R* genes during all rice growth stages and exhibited resistance to 10 Philippine races of BLB (Zhang, 2009).

Rice blast (caused by *Magnaporthe oryzae*) is another destructive disease affecting leaves, nodes, collar, panicles, and panicle neck during vegetative to reproductive stage causing 70–80% yield losses under severe infestation conditions (Zhu et al., 2005). More than 100 blast resistance genes have been identified; however, only 30 of them have been cloned and functionally characterized to develop linked markers for their effective use to provide resistance in cost‐effective manner against this fungal disease (Wang, Ebbole, & Wang, 2017; Zhao et al., 2018). One of the broad‐spectrum blast resistant genes, *Pi9* encoding nucleotide‐binding site–leucine‐rich repeat gene clusters, was found resistant to many of the blast races in different countries (Qu et al., 2006). The other blast resistance gene, *Pita2* mapped on the short arm of chromosome 12, confers broader resistance and has proven to be of high value in rice breeding for blast disease (Meng et al., 2020).

Rice cultivation is affected significantly by attack of various insect pests. Among the key insect pest in rice, brown plant hopper (*Nilaparvata lugens*), is a notorious insect pest causing not only severe yield losses across Asia but is also responsible for transmitting virus diseases such as rice grassy stunt virus (Cabauatan, Cabunagan, & Choi, 2009; Normile, 2008). To date, 37 brown plant hopper (BPH) resistance genes have been reported from cultivated rice and wild *Oryza* species (Li, Chen, Yin, Wang, & Chen, 2019; Wang et al., 2018; Yang et al., 2019). Among those, 20 genes were fine mapped and only eight genes (*Bph3*, *Bph14*, *Bph17*, *Bph18*, *Bph26*, *Bph29*, *Bph9*, and *Bph 32*) have been cloned and functionally characterized and are available for use in marker‐assisted introgression programs (Liu et al., 2015; Lv et al., 2014; Ren et al., 2016; Sun, Su, Wang, Zhai, & Wan, 2005, Qiu, Guo, Jing, Zhu, & He, 2012; Wang et al., 2015).The genes *Bph3* and *Bph17* were first reported in the Srilankan rice cultivar Rathu Heenati (Lakshminarayana & Khush, 1977) and found effective against four BPH biotypes and is reported to provide resistance to BPH even after 30 yr of deployment in the Philippines (Cruz, Arida, Heong, & Horgan, 2011). The cloning and functional characterization of *Bph3* and *Bph17* provides opportunity to effectively use these two genes in marker‐assisted breeding or gene pyramiding (Liu et al., 2015). The genes *Bph3 and Bph17* were also targeted for introgression in our study to obtain resistance against BPH. The Asian gall midge (GM) (*Orseolia oryzae*) is a serious insect pest of rice that is prevalent mainly in the wet season (WS) with an estimated annual yield loss of $550 million in different countries in Asia (Biradar et al., 2004). Till now, genetic studies have identified 11 major resistance (*R*) genes providing resistance to seven biotypes of the GM of rice prevailing mostly in southern Asian countries (Bentur et al., 2016; Divya, Himabindu, Nair, & Bentur, 2015; Himabindu, Suneetha, Sama, & Bentur, 2010; Kumar et al., 2005). Four GM resistance genes, designated as *Gm1*, *Gm2(NB‐ARC)*, *gm3(NB‐ARC)*, and *Gm4(NB‐LRR)*, have been functionally validated and linked markers are available for introgression (Bentur et al., 2016; Suvendhu et al., 2014; Sama et al., 2014; Sundaram, 2007). The *Gm4* gene used in the current introgression program confers resistance to Gm biotypes 1, 2, 3, 4, and 4M (Bentur et al., 2011). The *Gm4* gene encodes leucine rice repeat domain and exhibit hypersensitive type reaction where death of host cell occurs at the site of insect attack (Bentur et al., 2011; Divya et al., 2015). Pyramiding of GM resistance genes (*Gm1*, *Gm4*) and (*Gm4*, *Gm8*) was attempted through marker‐assisted breeding approach into the elite background of Improved Samba Mahsuri and an elite rice hybrid DRRH3, respectively (Divya et al., 2015; Kumar et al., 2017). In recent years, some efforts have been made to improve some elite rice varieties by marker‐assisted pyramiding of various genes conferring to GM, blast, and BLB resistance along with *Saltol* QTL for salinity tolerance (Das & Rao., 2015; Das, Rao, Varier, Prakash, & Prasad, 2018).

Keeping in view the multiple challenges faced by rice in rainfed environments under the present scenario of climate change, the present study was undertaken to develop high‐yielding resilient rice through stacking of multiple genes and QTL that can withstand in a cascade of multiple stresses including both abiotic as well as biotic stresses occurring regularly in rainfed environments.

## 2 MATERIAL AND METHODS

### 2.1 Plant materials

A high‐yielding genetic background line IR 91648‐B‐1‐B‐3‐1(hereafter named as IR91648) was used to develop multiple‐stress‐tolerant climate‐resilient rice. This breeding line was developed at IRRI from the cross of Moroberekan (ACC 12048)/3 × Swarna. We had used eight donor parents to introgress the targeted QTL and genes into the genetic background of IR91648. All the donors used in the crossing for their targeted QTL and genes are listed in Table 1.

**TABLE 1:** Abiotic and biotic stress‐tolerant donors used in development of multiple stress‐tolerant rice lines

Trait	Donor	Quantitative trait locus or gene	Reference
Drought plus submergence	IR96322‐34‐223‐B	*qDTY_1.1_*, *qDTY_2.1_*, *qDTY_3.1_*, *Sub1*	Sandhu et al., 2019
Drought	IR74371‐46‐1‐1	*qDTY_12.1_*	Bernier et al., 2007
Blast	WHD‐1S‐75‐1‐127	*Pi9*	Qu et al., 2006
Blast	‘Tadukan’	*Pita2*	Koide et al., 2009
Bacterial leaf blight	IRBB60	*Xa4*, *xa5*, *xa13*, *Xa21*	Song et al., 1997
Bacterial leaf blight	IRBB23	*Xa23*	Ona et al., 2010
Gall midge	‘Abhaya’	*Gm4*	Sun et al., 2005
Brown plant hopper	‘Rathu Heenathi’	*Bph3*, *Bph17*	Jairin, Phengrat, Teangdeerith, Vanavichit, and Toojinda, 2007

### 2.2 Crossing strategy

**FIGURE 1:**
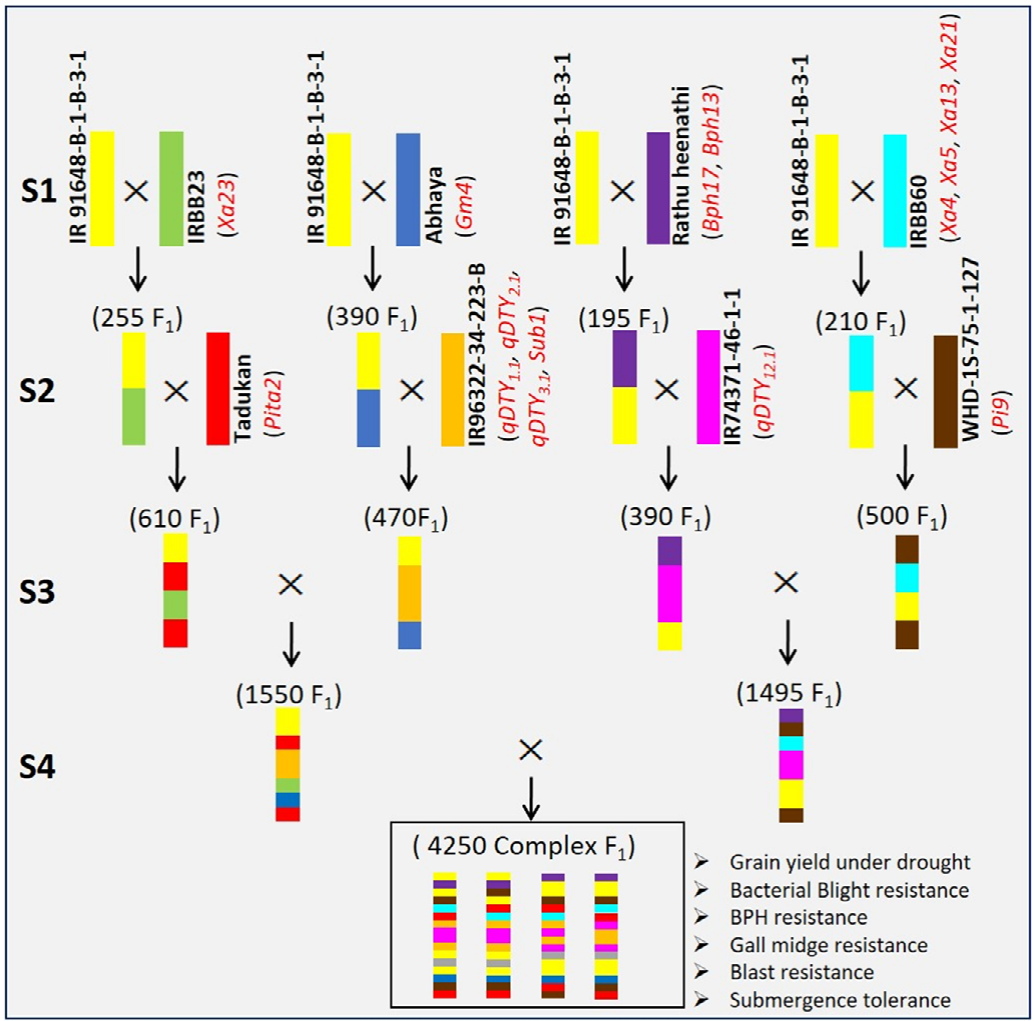
Assemble first (complex) crossing scheme to assemble targeted quantitative trait loci and genes into a high‐yielding breeding line (IR 91648‐B‐1‐B‐3‐1)

The assembled first‐crossing scheme (complex cross) was performed to assemble all the targeted QTL and genes into the high‐yielding breeding line IR 91648 using eight donors. The detailed crossing scheme with parents involved in individual crossing and number of F_1_ seeds produced from such crosses are presented in Figure 1. In brief, four crosses using parents IRBB23, Abhaya, Rathu Heenathi, and IRBB60 individually with IR 91648 in the first season and four F_1_ products were crossed with the remaining four donors (‘Tadukan’, IR 96322, IR74371‐46‐1‐1, and WHD‐1S‐75‐1‐127) in the second season. In further seasons (third and fourth), the F_1_ products were intercrossed strategically to develop a complex F_1_ population having the segments of all the donors used in crossing (Figure 1). The F_1_ products were confirmed for the desired alleles of respective donors using gene‐based linked markers to the respective QTL and genes in each season (Table 2). The generated complex F_1_ population carrying targeted QTL and genes were selfed through the F_6_ generation in order to get homozygosity.

**TABLE 2:** List of gene‐based linked markers and single nucleotide polymorphisms (SNPs) deployed in the study for development of climate‐resilient lines

Trait	Donor or pyramided line used	QTL or gene	SSR used	SNPs used (favorable allele)	Insertion–deletion marker	Gene‐based marker
Drought plus submergence	IR96322‐34‐223‐B	*qDTY_1.1_*	RM431, RM11943, RM12233 (linked markers)	snpOS0071 (A), snpOS0074 (G)	–	–
		*qDTY_2.1_*	RM324, RM3549, RM12868, RM12987, RM12995 (linked markers)	snpOS0078 (A), snpOS0079 (A)	–	–
		*qDTY_3.1_*	RM520, RM16030, RM416 (linked markers)	snpOS0085 (G), snpOS0089 (C)	–	–
		*Sub1*	–	snpOS0040 (T)	ART5	–
Drought	IR74371‐46‐1‐1	*qDTY_12.1_*	RM28099, RM28166, Indel 8 (linked markers)	snpOS00483(G), snpOS00484(A),	–	–
Blast	WHD‐1S‐75‐1‐127	*Pi9*	–	M891 (C)	MSU7_6_10381500 (M492, M493)	Pi9‐659T, Pi9‐1477G
Blast	‘Tadukan’	*Pita2*	–	snpOS00488(G)	MSU7_12_9177624 (M535, M536)	YL155/YL87, YL153/YL154
Bacterial leaf blight	IRBB60	*Xa4*	–	–	–	MP1, MP2
		*xa5*	–	snpOS0054 (AG)	–	Xa5S, Xa5R
		*xa13*	–	–	*xa13*‐promoter (M478Lm, M479Lm, M480Lm)	*xa13*F_130‐147/*xa13* R_1678‐1662
		*Xa21*	–	snpOS0061 (C)	Xa21s_exon (M769, M770)	U1/I1
Bacterial leaf blight	IRBB23	*Xa23*	–	M1207 (T)	–	Lj74
Gall midge	‘Abhaya’	*Gm4*	–	–	–	LLR‐del
Brown plant hoppers	‘Rathu Heenathi’	*Bph3*	RM586, RM589, RM190, RM7639, RM19311 (linked markers)	M899 (C)	–	–
		*Bph17*	RM401, RM8213 (linked markers)	M1113 (A)	–	–

### 2.3 Screening under reproductive‐stage drought stress

Drought screening in the WS was conducted in a rain‐out shelter facility at IRRI, while the dry season (DS) experiment was performed directly in the field of IRRI, Los Baños, during the years of 2018 and 2019. Screening for reproductive‐stage (RS) drought was performed using standardized protocol developed at IRRI using an appropriate breeding design (Kumar et al., 2009, 2014; Vikram et al., 2011). The experiment was laid out in alpha‐lattice design with two replications in plot size of 5 m^2^. Twenty‐one‐day old seedlings were transplanted in the main field. Recommended doses of nitrogen, phosphorus, and potassium was applied at the rate of 120:30:30 kg ha^−1^. Two doses of N fertilizer were applied at 10 and 30 d after transplanting before initiating the stress, and a third dose was applied along with life‐saving irrigation. Normal irrigation was maintained until 30 d after transplanting and excess water was drained out to initiate drought stress until the crop maturity. Perforated PVC pipes were installed in soil up to 1 m depth and 10 cm above the soil surface at regular places across the field to measure the water table. A short flash flooding was provided as a life‐saving irrigation when the water table was measured <1 m and all the susceptible checks started showing severe leaf rolling. Life‐ saving irrigation was provided and the field was drained out immediately after 8 h to start a new cycle of water stress.

### 2.4 Screening for biotic stress tolerance

Ninety‐five improved lines carrying various combinations of BLB genes (*Xa*) were inoculated under natural conditions in the field at IRRI, Philippines. Lines were inoculated 45 d after transplanting in the field using two isolates (*PXO61* and *PXO86*) of the BLB pathogen recommended at IRRI along with checks. The five uppermost leaves were inoculated in a single plant randomly chosen from each improved line in both the replications, and disease reaction was scored at 14 d after inoculation. Most of the BLB *R* genes confer resistance against two Philippines strains, *PXO61* (race1) and *PXO86* (race2) (Nino‐Liu, Ronald, & Bogdanove, 2006; Verdier, Cruz, & Leach, 2011). IR24 was included as one of the susceptible checks to most of the races of *Xoo* for reliable observations. The upper three leaves from three randomly chosen plants (total nine leaves) of each test line were clip‐inoculated with BLB suspension of ∼10^9^ cells ml^−1^ in concentration. Phenotypic reactions of the lines were recorded at 14 d after inoculation by measuring the lesion length, and lines were classified based on the standard evaluation system (SES) scale (IRRI, 2002).

Screening for leaf blast was performed in the uniform blast nursery of IRRI, where each test entries was sown in two rows with a 10‐cm distance between consecutive rows and three checks (CO‐39, highly susceptible; IR50, intermediate susceptibility; and IR 442‐2‐58, highly resistant check) were repeated after every 20 test entries across the nursery bed. The entire nursery bed was surrounded by two rows of C0‐39 as a disease spreader row. Disease scoring was recorded using SES scale after 15–20 d depending on the severity of leaf blast (IRRI, 2002).

The same 95 improved lines were screened against GM at the Bangladesh Rice Research Institute (BRRI) using a controlled glass house facility developed for GM screening. Lines were grown using Yoshida culture solution and sprouted seeds of test entries were sown separately in 11‐cm‐long lines in a row keeping 6.5 cm distance from line to line. Each line represents a test entry. Nine test entries, including one susceptible (BRRI dhan49) and one resistant (BRRI dhan33) check, were assigned separately in a row. Thus, a total of nine entries were grown in a cork sheet (58 by 45 cm) following complete randomized design with three replications. The seedlings were grown for 13–15 d. Approximately 100 mated GM females were released in a confined net at 15‐d after planting and allowed lay eggs on the test materials. During infestation, controlled temperature (27–32 °C) and relative humidity (85–90%) were maintained. Data on infested (‘onion shoots’ or silver shoots) and uninfested tillers on the test entries were recorded. Scores were made from the percentage infestations according to the SES (IRRI, 2002).

### 2.5 Grain quality testing

Homozygous lines possessing various QTL and gene combinations were analyzed for grain quality parameters at IRRI Grain Quality and Nutrition Center lab facility. The physiochemical grain properties, that is, grain length, grain width, percentage head rice recovery (HRR), chalkiness, amylose content (AC), and gelatinization temperature (GT) were measured in the present study. Harvested paddy grains from individual plots in replicated experiments were forwarded to a control drying facility at the IRRI Zeigler Experiment Station for bringing the moisture content up to 12–14%. Approximately 250 g of samples of properly dried paddy at 12–14% moisture content were dehulled using a dehuller (Satake Corporation) and milled further using Grainman mill (Grainman 60‐230‐60‐2AT) to separate head or whole grain from broken ones in order to measure the percentage HRR. Grain length, width, and chalkiness of milled rice were analyzed using the SeedCount SC5000 image analyzer (Next Instruments). Chalky grains having opaque or chalky areas that prevent the transmission of scattered light (Tashiro & Wardlaw, 1991; Yoshioka, Iwata, Tabata, Ninomiya, & Ohsawa, 2007). Grain shape was characterized on basis of length‐to‐width (L/W) ratio: slender, >3.0; medium, 2.1–3.0; bold, 1.1–2.0. Chalkiness, a highly undesirable grain quality parameter, was classified as follows: none (0%), small (<10%), medium (10–20%), and large (>20%). According to the IRRI (2002), the following scale has been used to classify the milled rice grain into various size and shape characteristics: extra long, >7.50 mm; long, 6.61–7.50 mm; medium, 5.51–6.60 mm; and short, <5.50 mm. Amylose content was measured on the basis of calorimetric analysis of amylose–iodine complex using the method of ISO 6647 (ISO, 2007). Absorbance of the samples for amylose–iodine complex at a wavelength of 620 nm was measured, and AC was quantified using the standard curve for known AC values. The detailed procedure was followed as previously described (Cuevas, Domingo, & Sreenivasulu, 2018). On the basis of AC content, milled rice can be grouped into following classes: waxy, 0–2%; very low, 3–9%; low, 10–19%; intermediate, 20–25%, and high, >25% (IRRI, 2002). Gelatinization temperature was measured through differential scanning calorimetry using the similar protocol described earlier by Cuevas et al. (2010). Samples were classified on the basis of GT observations as follows: low, <67 °C; intermediate, 68–73 °C; and high, ≥74 °C (Cuevas et al., 2010; Musyoki et al., 2015).

### 2.6 Genotyping

Presence of polymorphism between IR 91648 (introgression background) and all the donor parents, that is, IR 96322 (carrying *qDTY_1.1_*, *qDTY_2.1_*, *qDTY_3.1_*, *Sub1*), IR74371‐46‐1‐1 (*qDTY_12.1_*), WHD‐1S‐75‐1‐127 (*Pi9*), Tadukan (*Pita2*), IRBB60 (*Xa4*, *xa5*, *xa13*, *Xa21*), IRBB23 (*Xa23*), Rathu Heenati (*Bph3*, *Bph17*), and Abhaya (*Gm4*), were confirmed using their gene‐based linked markers reported earlier for the targeted QTL and genes (Table 2). Genomic DNA extraction from the leaves of 21‐d‐old seedlings was performed at the Genotyping Service Laboratory, IRRI, using CTAB method (Murray & Thompson, 1980). Polymerase chain reaction (PCR) amplification for targeted makers was carried out to confirm the presence of introgressed loci, and PCR products were resolved casting high‐resolution 8% (v/v) polyacrylamide gel electrophoresis (CBS scientific, model MGV‐202‐33) and running in a 1× TBE buffer at 90 v for 1.5–2 h depending on the product size. The separated DNA fragments after electrophoresis were stained with DNA gel stain (SYBR Safe) and visualized under an ultra‐violet trans‐illuminator (AlphaImager System). Genotyping of selected single plants in each generation from F_1_ to F_6_ was performed for targeted QTL and genes until achieving the homozygosity. The complex F_1_ population and homozygous lines (F_6_) were also genotyped with some trait‐based single nucleotide polymorphism (SNP) markers developed by IRRI using Kompetitive allele specific PCR SNP assay with Intertek as a service provider. The SNP markers linked to the traits such as snpOS0071 and snpOS0074 (*qDTY_1.1_*), snpOS0078 and snpOS0079 (*qDTY_2.1_*), snpOS0085 and snpOS0089 (*qDTY_3.1_*), snpOS00483 and snpOS00484 (*qDTY_12.1_*), snpOS0040 (*Sub1*), snpOS0054 (*xa5*), snpOS0061 (*Xa21*), and snpOS0006 (*Pita‐2*) were used for genotyping (https://gsl.irri.org/). The SNP markers linked to the *Bph3*, *Bph17*, and *Pi9* genes were genotyped using in house SNP genotyping platform available at IRRI (Table 2).

### 2.7 Data collection and statistical analysis

Data were recorded on days to flowering (DTF), plant height (PH), and GY under nonstress and drought conditions. Days to flowering was calculated from the days to seeding to the 50% of flowering in plot. The PH (cm) was measured from the soil surface to the tip of the plants and was taken as the average from the three plants. Plants were harvested at physiological maturity, oven‐dried, and adjusted at 12% moisture content to measure GY (Venuprasad et al., 2009).

The phenotypic data obtained from the experiments were analyzed for the computation of trial means, standard error of difference, and broad‐sense heritability (*H* ^2^) using PB Tools v1.4 developed at IRRI (http://bbi.irri.org/products). Least significant difference (LSD) at *P* =.05 significance was used to compare the means of improved lines and to infer the significant differences of the traits studied between the entries. Single‐trial analyses were conducted using a linear mixed model that considered genotype factor as a fixed effect and replicate and block within replicate effects as random,

The model used for the alpha‐lattice design was as follows:

$$Y_{ijk}\;=\;\mu \;+\;G_{i}\;+\;Rj\;+\;BK\;\left(R_{j}\right)\;+\;e_{ijk}$$

where, *Y_ijk_* is measurement recorded in plot, μ is overall mean, *G_i_* is the effect of *i*th genotype, *R_j_* is the effect of the *j*th replicate, BK(*R_j_*) is the block effect of *j*th replicate, and *e_ijk_* is the error.

The model used for the augmented randomized complete block design was as follows:

$$Y_{ijk}\;=\;M+G_{i}+B_{l}+E_{ilk}$$

where, *Y_ijk_* is the measurement recorded in plot, *M* is the overall mean of plot, *G_i_* is the effect of the *i*th genotype, *B_l_* is the effect of the *l*th block, and *E_ilk_* is the experimental error.

To estimate *H* ^2^, variance components were computed by considering all the factors including genotypes as random; *H* ^2^ was estimated as follows:

$$H\;\;=\;{\sigma^{2}}_{G}/\left({\sigma^{2}}_{G}\;+{\sigma^{2}}_{E}/r\right)$$

Where H is broad sense heritability, σ^2^_G_ represents the genetic variance, σ^2^_E_ the error variance and r the number of replications.

## 3 RESULTS

### 3.1 Genomics‐assisted breeding for drought, submergence, blast, BLB, GM, and BPH traits

A breeding strategy involving genotypic and phenotypic selection approach at each generation was implemented in assembling and fixing of all the targeted QTL and genes in the study. In total, 4,250 complex F_1_ seeds were generated by strategic crossing using eight donors and a high‐yielding breeding line, IR 91648, using marker‐assisted foreground selection with known QTL and genes. The crossing scheme and number of seeds generated from each cross attempted in developing the complex F_1_ populations is presented in Figure 1.

**FIGURE 2:**
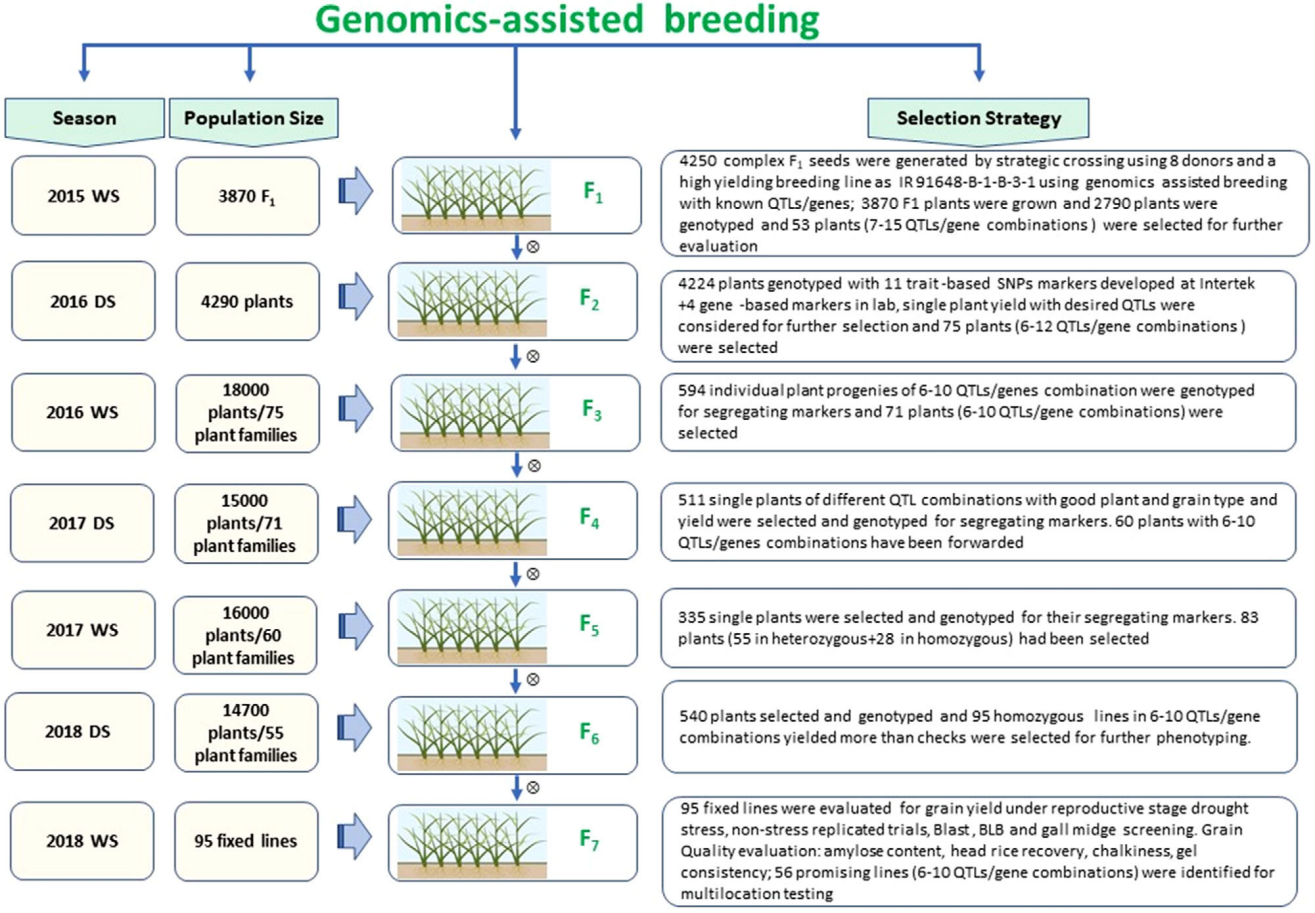
Genomics‐assisted forward breeding selection strategy

In total, 3,870 F_1_ plants were grown in the field, 2,790 individual plants harboring targeted QTL and gene combinations were screened for their respective markers and genes, and 53 plants in 7–15 QTL and gene combinations were selected and harvested individually. From the 53 selected plants, 4,290 F_2_ individual plant progenies were grown in the field, and leaves of 4,224 healthy plants were collected for genotyping with Intertek (for 11 SNP markers) as well as in‐lab screening (for four gene‐based markers). In total, 4,144 plants were scored using genotyping data with available SNP markers linked to the traits such as *qDTY_1.1_*, *qDTY_2.1_*, *qDTY_3.1_*, *qDTY_12.1_*, *Sub1*, *xa5*, *Xa21*, *Pita‐2*, *Pi9*, *Bph3*, and *Bph17* from the Intertek service provider. Gene‐based markers were used for screening of the remaining four genes (*Xa4*, *xa13*, *Xa23*, and *Gm4*) targeted in the present study. Based on genotypic data and single‐plant yield data, a total of 75 F_2_ plant families in 6–12 QTL and gene combinations with GY higher than checks (‘MTU1010’, IR64, and IR 915468) were selected for evaluation in subsequent generations until F_6_. The total number of plants evaluated in the field, genotyped ,and selected in each generation is shown in Table 3. The large population size was maintained in each generation to get the desired recombinants available for selection without having any negative interaction for plant yield within same QTL and gene combinations. The plants in homozygous condition with GY higher than or similar to checks were selected, and finally, 95 homozygous F_6_ lines carrying 6–10 QTL and genes were forwarded for further phenotypic evaluation under nonstress, RS drought, biotic screening, and grain quality testing. Details on the foreground selection, phenotyping, and the number of plants selected in each generation using a genomics‐assisted breeding approach are presented in Figure 2.

**TABLE 3:** Number of plants carrying multiple quantitative trait loci (QTL) or genes evaluated in field, genotyped, and selected in each generation

Generation	Total no. of plants evaluated in the field	Total no. of plants genotyped (trait‐based SNPs from Intertek or linked or gene‐based PCR markers in lab)^a^	No. of selected plants (based on genotyping and field evaluation)	No. of selected plants in QTL or gene combinations						
				6	7	8	9	10	11	12
F_1_^b^	3,870	2,790 (linked or gene‐based markers)	53	–	4	4	13	10	7	5
F_2_	4,290 (from 53 plant families)	4,224 (11 trait‐based SNPs, four gene‐based markers)	75	15	20	15	10	7	5	3
F_3_	18,000 (from 75 plant families)	594 (linked or gene‐based markers)	71	11	19	20	17	8	–	–
F_4_	15,000 (from 71 plant families)	511 (linked or gene‐based markers)	60	7	20	21	6	6	–	–
F_5_	16,000 (from 60 plant families)	335 (linked or gene‐based markers)	83 (55 in heterozygous, 28 in homozygous)	6	18	31	19	9	–	–
F_6_	14,700 (from 55 plant families)	540 (linked or gene‐based markers)	95 (homozygous lines)	11	33	32	7	12	–	–
F_7_	56 promising lines (from 95 homozygous lines)	56 (11 trait‐based SNPs, four gene‐based markers)	–	3	8	15	18	12	–	–

^a^ SNP, single nucleotide polymorphism; PCR, polymerase chain reaction.

**^b^** In F_1_, we also had the combination of 13 QTL or genes in three plants, 14 QTL or genes in five plants, and 15 QTL or genes in two plants.

### 3.2 Evaluation of introgressed lines under nonstress and RS conditions

The homozygous improved lines carrying 6–10 QTL and gene combinations were evaluated under nonstress and RS conditions. In total, 28 entries carrying multiple QTL and genes were fixed in the F_5_ generation and were evaluated under nonstress and RS conditions (Supplemental Figure S1). Mean DTF, PH, GY, *H* ^2^, population range, and LSD_0.05_ of homozygous improved and checks under nonstress and RS conditions are presented in Table 4. The percentage GY advantage of improved lines over the popular checks, namely IR64 and Swarna, was computed as 10 and 14%, respectively, under nonstress conditions. Improved line IR18L1059 with two drought QTL (*qDTY_1.1_*, *qDTY_12.1_*) yielded least among the evaluated lines, while IR18L1016 with four drought QTL (*qDTY_1.1_*, *qDTY_2.1_*, *qDTY_3.1_*, *qDTY_12.1_*) produced maximum GY (2,854 kg ha^−1^) under drought stress situation.

**TABLE 4:** Mean performances for days to flowering, plant height, and grain yield of homozygous improved lines compared with checks under nonstress (NS) and reproductive‐stage (RS) drought conditions

No. of improved lines evaluated	Generation	Experimental design	Season^a^	Environment	Stress level	Days to flowering^b^						Plant height						Grain yield					
						IB^c^	Check_1_	Check_2_	TM	LSD_0.05_	*H* ^2^	IB	Check_1_	Check_2_	TM	LSD_0.05_	*H* ^2^	IB	Check_1_	Check_2_	TM	LSD_0.05_	*H* ^2^
						d						cm						kg ha^−1^					
28	F_5_	Alpha lattice	2018DS	NS	–	90	88	105	90	6	.77	115	107	88	101	9.1	.68	6,300	5,500	5,230	6,075	1,190	.46
28	F_5_	Alpha lattice	2018DS	RS	Moderate	79	77	–	81	4	.89	83	84	64	77	8	.76	735	464	NF^d^	1,649	1,040	.51
95	F_6_	Alpha lattice	2018WS	NS	–	92	83	106	89	3.34	.94	117	108	85	102	12.7	.73	5,545	4,168	3,970	3,743	1,554.5	.54
95	F_6_	Alpha lattice	2018WS	RS	Severe	91	82	–	84	8	.8	88	93	80	88	21	.35	490	269	NF	745	786	.49
56	F_7_	Augmented RCBD^e^	2019DS	NS	–	82	80	102	86	4	.92	101	107	75	103	8	.78	7,351	6,974	6,200	7,318	1,100	.82

^a^DS, dry season; WS, wet season.

^b^Check_1_, ‘IR64’; Check_2_, ‘Swarna’; TM, trial mean of improved lines; *H* ^2^, broad‐sense heritability.

^c^IB, introgression background (IR 91648‐B‐1‐B‐3‐1).

^d^NF, not flowered.

^e^RCBD, randomized complete block design.

Ninety‐five improved F_6_ lines were screened under nonstress and RS drought conditions in 2018WS along with biotic stress screening and grain quality testing. The analyzed mean value for DTF, PH, and GY along with the LSD and *H* ^2^ value under nonstress and RS conditions are represented in Table 4. Further, 56 F_7_ promising lines were evaluated in 2019DS under nonstress condition, and the analyzed mean value for DTF, PH, and GY along with LSD and *H* ^2^ is presented in Table 4.

### 3.3 Bioassays

#### 3.3.1 Bacterial leaf blight

The presence of a high level of resistance against BLB was confirmed among the tested improved lines pyramided with multiple QTL and genes. Out of 95 improved lines evaluated, the only single line, namely IR18L1021, showed susceptibility for BLB, while four lines showed moderate level of resistance (Supplemental Table S1). Excluding these five lines, the remaining improved lines showed a high level of resistance against this disease, with score of 1 to 3 evaluated following the IRRI (2002) SES. The high‐yielding breeding line (IR 91648) used in introgression showed susceptible reaction (score 7) against the BLB.

#### 3.3.2 Blast

Ninety‐five homozygous improved lines were also screened against blast disease at IRRI natural blast screening facility using a natural population of mixed isolates of blast disease. Blast donors WHD‐1S‐75‐1‐127 and Tadukan used in introgression were found very effective against blast disease and showed resistant reaction with a score of 0 (highly resistant). The majority of improved lines exhibited complete resistance against blast when compared with the susceptible control lines such as CO39 and IR72 (Supplemental Figure S2; Supplemental Table S1). In total, 75 improved lines showed resistance reaction (score 0), while 18 lines expressed resistance against blast (score 1–3). The high‐yielding breeding line IR 91648 used as recurrent parent has shown highly susceptible reaction (score 9) against blast disease.

#### 3.3.3 Gall midge

Ninety‐five improved lines were screened against GM at BRRI using a controlled glass house facility. Out of 50 improved lines carrying *Gm4* gene in combination of other QTL and genes, eight lines showed complete resistance, while the remaining 42 lines exhibited moderate reaction against GM. Data on infested (‘onion shoots’ or silver shoots) and uninfested tillers revealed seven lines with *Gm4* shown HR reaction (0% infestation), while one line displayed resistance reaction (2.22%) (Supplemental Table S2). Thirty lines carrying other gene combinations, without any of the GM resistance genes as expected, showed complete susceptibility against GM similar to susceptible control BRRI dhan49 (Supplemental Figure S3).

### 3.4 Product profiling with morphological and grain quality data

A total of 95 homozygous improved lines carrying combinations of QTL and genes were characterized for grain and cooking quality parameters using the harvested seeds from the F_6_ generation. Grain length of improved lines varied from 5.9 to 7.19 mm among the improved lines with average mean value of 6.7 ± 0.4 mm. Grain width of milled rice ranged from 2.12 to 2.48 mm, with average mean value of 2.3 mm and standard deviation (SD) of 0.1 mm (2.3 ± 0.1 mm) (Supplemental Table S3). Grain length and L/W ratio of improved lines was observed as 6.7 mm and 3.1, respectively, indicating most of the improved lines (56 lines) evaluated in the study had long, slender grain type. We also found 39 lines with medium slender grain type with 2.38–2.99 in L/W ratio. The percentage HRR ranged from 30.6 to 66.85% among the improved lines and 20 lines showed >60% HRR (Supplemental Table S3) vs. 53, 65, and 45% of MTU1010, Swarna, and IR64 checks, respectively. Chalkiness in improved lines varied from 0.4 to 22.8% with mean value of 4.7%. Out of 95 lines evaluated, a total of seven lines showed chalkiness >10% and were not carried forward to the F_7_ generation for further evaluation. Most of the lines in this study showed intermediate GT content. All improved lines showed AC in the range of 18.8–26.45%. A total of 18 lines have AC < 20 (low amylose), 72 lines 20–25 (intermediate), and five lines measured higher AC of >25 (Supplemental Table S3). All these physiochemical properties of rice grains were also analyzed for some popular checks of rice (MTU 1010, Swarna, IR64, and IR 74371‐70‐1‐1) and high‐yielding background (IR 91648) used in introgression through improved and data is provided in Supplemental Table S3. The developed improved lines carrying six to 10 QTL and genes for abiotic and biotic stress and superior grain quality trait characteristics were found promising to carry forward for further testing and release. Fifty‐six such promising lines were selected based on higher GY under nonstress as well as RS drought, resistant to blast and BLB, and having superior grain quality traits. Lines were categorized for further evaluation in the targeted environment based on AC and other grain quality preferences of rice consumers of a country. Supplemental Table S4 represents an example of multiple‐stress‐tolerant lines with desired grain quality characteristics for various countries of southern and southeastern Asia, and these lines can be evaluated further as yield trial evaluation.

### 3.5 Promising lines and their yield performances over popular checks

**FIGURE 3:**
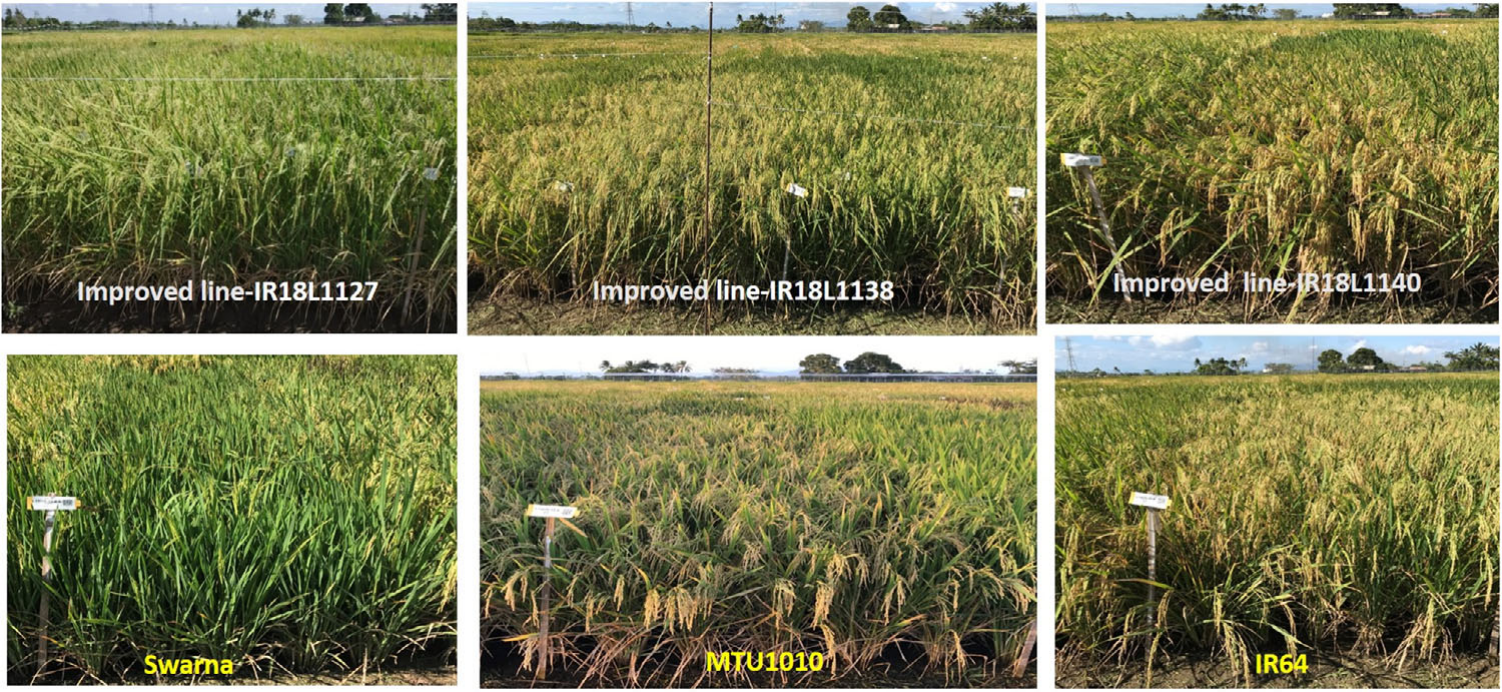
Improved lines carrying multiple quantitative trait loci and genes in comparison with popular varieties of rice

Lines carrying multiple QTL and genes performed well for GY compared with checks under nonstress and RS conditions and showed resistance against blast, BLB, and GM with desired grain quality characteristics were selected as promising lines for further evaluation in targeted environments. A total of 10 such promising lines carrying six to 10 QTL and genes for various abiotic as well as biotic stresses are shown in Table 5, which can be further evaluated in multiple environments. An improved line, namely IR18L1156 carrying 10 QTL and genes (*qDTY_1.1_*, *qDTY_12.1_*, *Sub1*, *Pi9*, *GM4*, *Xa4*, *xa5*, *Xa21*, *Xa23*, *Bph3*) showed yield advantage of 3.5 and 12.7% over IR64 under nonstress condition in 2018WS and 2019DS, respectively, and also exhibited phenotypically high level of tolerance or resistance to abiotic and biotic stresses with good milling and cooking quality (Table 5). Similarly, line IR18L1149 carrying seven QTL and genes (*qDTY_1.1_*, *qDTY_2.1_*, *qDTY_3.1_*, *Sub1*, *Pi9*, *Xa21*, *Xa23)* showed yield advantage of 26.8 and 6.1% over the IR64 in 2018WS and 2019DS, respectively. Field performances of some of multiple‐stress‐tolerant lines compared with popular checks of rice are also presented in Figure 3. Promising lines showed a similar or higher GY over the checks rice varieties and performance of such lines can further be tested for yield stability by conducting multi‐location trials at their targeted environments.

**TABLE 5:** Performance of promising multiple stress‐tolerant improved lines over the popular check

			Grain yield											
			Under nonstress		Under reproductive‐stage drought stress		Score^a^						Yield increase over ‘IR64’	
Designation	Quantitative trait loci (QTL) or gene combination	No. of QTL or genes	2018 wet season	2019 dry season	Severe drought stress	Moderate drought stress	Bacterial leaf blight	Blast	Chalkiness	Amylose content	GT^b^	Percentage HRR^c^	Wet season	Dry season
			kg ha^−1^						%			%		
IR18L1140	*qDTY_1.1_*, *qDTY_2.1_*, *qDTY_3.1_*, *qDTY_12.1_*, *Sub1*, *xa5*	6	5,299	8,586	1,760	2,494	1	4	6.22	20.3	High	61.6	21.34	18.77
IR18L1127	*qDTY_1.1_*, *qDTY_2.1_*, *qDTY_3.1_*, *qDTY_12.1_*, *Sub1*, *GM4*, *Pi9*, *xa5*, *Xa21*, *Bph17*	10	512	7,086	953	1,425	1	1	1.86	21.4	Inter.	61.3	18.70	1.58
IR18L1138	*qDTY_1.1_*, *qDTY_3.1_*, *qDTY_12.1_*, *Sub1*, *Pi9*, *xa5*, *Xa21*, *Xa23*, *Bph3*	9	4,315	8,020	596	1,606	1	0	5.45	20.5	Inter.	61	3.5	13.40
IR18L1137	*qDTY_1.1_*, *qDTY_2.1_*, *qDTY_3.1_*, *qDTY_12.1_*, *Sub1*, *GM4*, *Pi9*, *Xa21*, *Xa23*, *Bph17*	10	5,130	7,964	955	1,883	1	1	1.86	21.4	Inter.	61.3	18.72	12.43
IR18L1149	*qDTY_1.1_*, *qDTY_2.1_*, *qDTY_3.1_*, *Sub1*, *Pi9*, *Xa21*, *Xa23*	7	5,698	7,427	705	1,777	1	0	6.53	25	Inter.	53.3	26.85	6.10
IR18L1150	*qDTY_1.1_*, *qDTY_2.1_*, *qDTY_3.1_*, *Sub1*, *Pi9*, *Xa21*, *Xa23*, *Bph3*	8	5,587	7,241	1,404	2,195	1	0	6.44	20.6	Inter.	54.6	25.4	3.69
IR18L1156	*qDTY_1.1_*, *qDTY_12.1_*, *Sub1*, *Pi9*, *Gm4*, *Xa4*, *xa5*, *Xa21*, *Xa23*, *Bph3*	10	4,322	7,988	1,005	2,002	1	0	4.89	21.3	High	61.4	3.56	12.70
IR18L1160	*qDTY_1.1_*, *qDTY_3.1_*, *qDTY_12.1_*, *Sub1*, *Pi9*, *xa5*, *Xa21*, *Xa23*, *Bph3*	9	4,315	7,716	596	1,532	1	0	5.45	20.5	Inter.	61	3.5	9.62
IR18L1165	*qDTY_1.1_*, *qDTY_3.1_*, *qDTY_12.1_*, *Sub1*, *Pi9*, *xa5*, *Xa21*, *Xa23*, *Bph3*	9	4,315	7,565	598	1,070	1	0	5.45	20.5	Inter.	61	3.5	7.81
IR18L1170	*qDTY_3.1_*, *qDTY_12.1_*, *Sub1*, *Pi9*, *xa5*, *Xa21*, *Xa23*, *Bph3*	8	4,276	8,445	966	1,446	1	3	5.77	20.5	Inter.	55.2	2.53	17.42
‘IR64’			4,168	6,974	210	760	–	–	9.37	19.7	High	45.8	–	–
‘Swarna’			3,970	6,200	0	230	–	–	0.78	25.2	Inter.	64.5	–	–
Mean			3,744	7,318	745	1,649	–	–	–	–	–	–	–	–
LSD_0.05_			1,555	1,100	786	1,040	–	–	–	–	–	–	–	–
Broad‐sense heritability (*H* ^2^)			.54	.82	.49	.51	–	–	–	–	–	–	–	–

^a^Bacterial leaf blight and blast score: 0, immune; 1, highly resistant; 3, resistant; 5, moderately resistant; 7, susceptible; 9, highly susceptible.

^b^GT, gelatinization temperature; Inter., intermediate.

^c^HRR, head rice recovery.

## 4 DISCUSSION

Grain yield with preferred grain quality as well as resistance or tolerance against existing and emerging biotic and abiotic stresses is needed to address the production constraints and recent environmental challenges faced by rice growers. Genomics‐assisted breeding with earlier identified gene‐based closely linked markers could be of great help to plant breeders in combining tolerance to multiple stresses together with preferred grain quality and yield potential. This is one of the first studies undertaken successfully in combining of up to 10 QTL and genes in a single background through marker‐assisted foreground selection that can provide tolerance or resistance to six targeted traits: drought, flood, blast, BLB, GM, and BPH.

Marker‐assisted breeding strategies in rice have been instrumental in transferring the major‐effect QTL and genes into mega varieties of rice and have proven successful in achieving the desired level of tolerance or resistance to various abiotic and biotic stresses because of presence of genomic regions contributing to large effects on the traits as against other cereals like wheat (*Triticum aestivum* L.) and maize (*Zea mays* L.) (Emebiri et al., 2017; Li et al., 2018; Qin et al., 2014; Zhang et al., 2017). In most of the previous efforts, pyramiding of multiple resistance genes I or QTL have been found promising for the elaboration of tolerance or resistance level of rice cultivars with higher yields. Marker‐assisted pyramiding of QTL for tolerance to abiotic stress traits such drought and submergence (Kumar et al., 2018; Sandhu et al., 2019; Septiningsih et al., 2015; Shamsudin et al., 2016; Swamy et al., 2013), resistance to blast (Fukuoka et al., 2015; Singh et al., 2013), BLB (Das et al., 2018; Pradhan et al., 2015; Suh et al., 2013), BPH (Jena, Hechanova, Verdeprado, Prahalada, & Kim, 2017; Wang et al., 2015), and GM (Divya, Singh, Nair, & Bentur, 2016) has been reported to achieve the expected improvement in resistance or tolerance in rice.

Four well‐proven drought QTL (*qDTY_1.1_*, *qDTY_2.1_*, *qDTY_3.1_*, and *qDTY_12.1_*) for GY under RS drought stress (Bernier, Kumar, Ramaiah, Spaner, & Atlin, 2007; Ghimire et al., 2012; Venuprasad et al., 2009; Vikram et al., 2011) have been used for current introgression programs, which can provide a yield advantage of 10–15% individually and >25% under RS drought stress when two or more such *qDTY* genes are combined together (Kumar et al., 2014, 2018). Despite being known to have good drought tolerance in traditional drought‐tolerant donors, characterization for yield potential, plant type, grain type, and eating quality are most important before its use in any breeding program (Kumar et al., 2014; Vikram et al., 2015). In this context, we had used an improved breeding line IR 96322 to pyramid *qDTY_1.1_*, *qDTY_2.1_*, *qDTY_3.1_*, and *Sub1* providing tolerance to both drought and flood with good grain type (medium to long slender) and improved plant type (medium height and lodging resistance) in the current study.

Submergence tolerance in rice is controlled by a well‐known flood‐tolerant *Sub1* gene, which can provide survival to rice lines up to 2 wk of complete submergence (Septiningsih et al., 2015; Xu & Mackill, 1996).The pyramiding of drought and submergence together is an important and needed breeding strategy for those rainfed lowland areas where flooding prevails during early crop stage and subsequently face drought at terminal stages in a single crop season. We had developed the improved lines such as IR18L1127 and IR18L1137 pyramided with four drought GY QTL (*qDTY_1.1_*, *qDTY_2.1_*, *qDTY* *_3.1_*, and *qDTY_12.1_*) and *Sub1* together, which will be very useful for cultivation in drought‐ and flood‐prone areas. The combined drought and flood‐tolerant rice varieties, namely CR Dhan 801, Bahuguni dhan‐1, and Bahuguni dhan‐2, have been developed using marker‐assisted introgression and released for cultivation in India and Nepal (Sandhu et al., 2019).

The ongoing developments in molecular markers with available low‐cost SNP chips has played an important role in accumulating favorable alleles more precisely, quickly, and effectively, particularly in pyramiding multiple complex traits and can maximize the expected genetic gain. In this context, a step‐wise breeding strategy that effectively combines marker‐assisted foreground selection and precise field selection has been deployed in implementation of the introgression program. The crossing program used in transferring the desirable alleles from eight different donors in this study was unique, where deployment of genomic tools such as well‐known markers and trait‐linked SNPs for high‐value QTL and genes had been used to track the desirable alleles in F_1_ populations, complex F_1_ populations, and in segregating materials of each generation until the homozygosity was achieved. The earlier‐known complex crossing programs in various crops, such as MAGIC (multi‐parent advanced generation intercross) in rice, had been developed for prebreeding schemes using the intercrossing of eight elite lines having high yield potential, good grain quality, and tolerance to a range of biotic and abiotic stresses; however, genotyping was performed later in the F_4_ stage in order to find known and novel major genes and QTL in the developed lines (Bandillo et al., 2013).

The strategy led to selection of the most appropriate plants with desired QTL and gene combinations in each segregating generation. The individual plants with the same QTL and gene combinations without observing any yield penalty compared with the popular checks, such as IR64, Swarna, and MTU1010, were selected for genotyping against presence of desired QTL and genes. A similar breeding strategy has been employed in selecting the most promising backcross introgression lines with desired QTL combination by marker‐assisted backcrossing combined with field phenotyping (Kumar et al., 2018; Sandhu et al., 2019; Shamsudin et al., 2016).

In our study, we had maintained a large population size (4,290–18,000 plants) in early generations for selecting most appropriate plants possessing the targeted QTL and gene combinations, desired plant type, and higher GY. Theoretically, it has been expected to maintain, on average, 800 F_2_ individual plants in order to get 50 lines with desired genotype fixed at two loci and frequency of desired homozygotes for two linked loci at the F_6_ generation (closure to fixation) will decrease nearly thrice compared with the F_2_ generation (Arbelaez et al., 2019). Keeping 18,000 F_3_ plants in the current study with six to 10 QTL and gene combinations has not only maximized the chances of getting positive interactions between QTL and genes but also led to an increase in capturing the hidden genetic variations. The number of plants selected in the field to genotype for presence of their respective QTL or gene has been shown in Figure 2. Nowadays, most of the plant breeding programs are deploying marker selection at very early generations, such as F_2_ onwards, in order to reduce the number of individuals to be genotyped as well as the cost of genotyping. The effectiveness of early generation selection in introgression of various drought QTL has been successfully demonstrated by Kumar et al. (2018) using genotyping and multi‐season phenotyping data of introgressed lines in various genetic backgrounds in rice.

Among biotic stress‐tolerant genes, we used three dominant (*Xa4*, *Xa21*, and *Xa23*) and two recessive (*xa5* and *xa13*) BLB resistance genes in introgression program, which can provide broad and durable resistance to our newly developed rice lines against BLB disease. Two improved lines (IR18L1135 and IR18L1144), which were not carrying any *R* genes for BLB, showed susceptible disease reactions similar to IR24 used as one of the highly susceptible check to most of the races of *Xoo*. Few of the improved lines, despite having a single recessive *R* gene *xa5*, showed resistant reaction similar to lines pyramided with two, three, or four resistant BLB gene combinations against the predominant virulent Philippines strain (*PXO61*). Many of the previous studies have documented the recessive gene *xa5* as one of the most effective *R* genes providing a broader level of resistance against many strains of *Xoo* (Garris, McCouch, & Kresovich, 2003; Huang et al., 2016; Jiang et al., 2006; Mishra et al., 2013). Most of the improved lines developed in the present study carry the *Xa21* gene in combinations of other *R* genes such as *Xa23* for BLB. The *Xa21* gene has been considered as the most effective BLB resistance gene and it has been widely used in introgression program against BLB in most of the rice‐growing countries of Asia (Cao, Zhan, Zhuang, & Cheng, 2005; Chen, Lin, Xu, & Zhang, 2000; Huang et al., 1999; Singh et al., 2001). A dominant resistance gene *Xa23*, identified from wild species of rice, confers an extremely broad level of resistance alone or in combination with other *R* genes to various *Xoo* strains isolated from different rice‐growing countries (Jiang et al., 2020; Wang et al., 2015). In the present study too, *Xa23* in combination with *Xa21* showed complete resistance against BLB disease.

Two blast resistance genes, *Pi9* and *Pita2*, were used in the current introgression in order to develop rice lines with multiple resistance and tolerances. Improved lines carrying either *Pita2* or *Pi9* alone showed complete resistance to blast disease. However, only two improved lines (IR18L1134 and IR18L1139) showed *Pita2* gene, while remaining all blast‐resistant lines were carrying *Pi9* gene. Several previous studies have reported that *Pi9* alone can provide broader and more durable resistance against rice blast disease similar to our current findings (Li et al., 2019). Improved lines were screened in a controlled glass house facility developed for GM screening at BRRI. Lines carrying *Gm4* showed complete resistance against GM disease ranged from 0 to 2.22% infestation; however, most of the lines, despite having the *Gm4* gene, showed moderate reaction against GM, suggesting some hidden interactions prevailing between *Gm4* with other introgressed QTL and genes. The role of such hidden epistatic influencing of the expression of a targeted phenotype has been well documented in various QTL mapping and marker‐assisted drought breeding program in rice (Sandhu et al., 2018; Yadav et al., 2019). Promising lines carrying BPH‐tolerant genes will be evaluated by testing in BPH hot spot areas during the conduct of multi‐location trials.

Furthermore, the developed improved lines were also evaluated for grain quality parameters, which have become a very crucial component for varietal development, release, and wider acceptance. Rice consumers have diverse preferences for grain quality that varies across countries and regions (Calingacion et al., 2014). We had succeeded in the breaking of unfavorable linkages and elimination of inferior plants with poor grain type during the selection process and identified the improved lines combining higher GY and good grain quality traits.

The developed improved lines were classified into different categories based on grain length, shape, and AC and data for 95 improved lines along with five checks of rice are presented in Supplemental Table S5. In the process of developing new varieties for release and commercial production, appearance of grain size and shape are critical and the first and foremost criteria for rice quality that breeders should consider in their selection (Rani, Pandey, Prasad, & Sudharshan, 2006).Various countries in southern and southeastern Asia have varied preferences particularly for grain length, shape, AC, and aroma (Calingacion et al., 2014). For instance, long, slender grains with intermediate GT and intermediate AC have been favorable preferences for countries like India, Nepal, Pakistan, and Malaysia, while high amylose with long and medium slender grains have been the choice of preference among consumers of Bangladesh, Myanmar, and Sri Lanka. Very low (waxy) with medium bold grains have been preferred in Lao People's Democratic Republic, and low AC with medium grain type has been preferred in Cambodia, parts of Thailand, China, and Taiwan (Calingacion et al., 2014; Cuevas, Pede, McKinley, Velarde, & Demont, 2016). We have developed improved lines carrying multiple QTL and genes for abiotic and biotic stresses with diverse grain quality combinations (Supplemental Table S6). For example, in the present study, 18 improved lines had long, slender grains with intermediate AC and GT and are recommended as promising lines to be targeted to countries such as India and Nepal for further evaluation and release through their national release system. A total of four improved lines with long and medium slender grains and high AC can be targeted for Bangladesh, Myanmar, and Sri Lanka for testing and release, while 18 lines with estimated low AC can be targeted to various countries of southeastern Asia.

Although our study demonstrated that unique genomics‐assisted breeding coupled with funnel mating design is a powerful tool to improve complex traits and for breeding climate‐resilient rice varieties, much more research is needed as the strategy will be more complex, and trade‐offs of different traits will need to be considered in application of such strategies that aim to combine a large number of QTL and genes. For instance, the present study started with aim to introgress 15 QTL and genes; however, we could develop plants containing a maximum of 10 QTL and genes in homozygous condition. Even though each of the 15 QTL and genes were successfully introgressed individually in one or the other plants as well as in the early generation, all QTL were successfully combined in heterozygous conditions, this study did not succeed in combining all 15 QTL and genes in one plant. This may have resulted because of negative interaction between some of these QTL and genes resulted in rejection of such plants possessing more than 10 genes and QTL because of their lower yield or inferior plant type in subsequent generations. Maintenance of larger population size may allow creation of higher recombination leading to possible breakage of such negative interactions, thus allowing selection of plants with more than 10 QTL and genes and better agronomic performance.

## 5 CONCLUSION

This study demonstrates the potential of genomic‐assisted breeding in successfully stacking multiple genes and QTL into a single rice line with both high yield and superior quality in less than 5 yr and increase the selection efficiency that led to successful varietal development, thereby enhancing genetic gain under normal conditions as well as abiotic and biotic stresses. The developed improved lines could be used as a line or variety to be released after multi‐location evaluation in national and provincial coordinated trials in their respective targeted countries. Lines developed in this study can also be used as a parental line in developing multiple‐stress‐tolerant varieties following simple crosses vs. the cumbersome process of using multiple parents to assemble and combine QTL and genes followed in the present study.

## AUTHORS' CONTRIBUTIONS

SY was involved in material development, genotyping, observations, plant selection, experimental data analysis, and drafting the manuscript; NS helped in genotyping and involved in critical revision of the manuscript; SD conducted the complex crossing phase of the study and helped in the revision of the manuscript; VKS was involved in critical revision of the manuscript; MC helped in genotyping; RRM & AR was involved in GM screening at Bangladesh; AK was involved in the design of the experiment, conceptualization of the result and in the critical revision of the manuscript. All authors approved the final version of the manuscript.

## ETHICAL STANDARDS

This study complied with the current law of the Philippines where this research work was conducted.

## CONFLICTS OF INTEREST

The authors declare that they have no conflicts of interest.

## Supporting information


**Supplemental Table S1** Disease Reaction of improved lines against BLB and Blast disease

**Supplemental Table S2** Gall midge resistant improved lines

**Supplemental Table S3** Grain quality data of improved lines for product profiling to deploy in targeted breeding

**Supplemental Table S4** Classification of improved lines based on desired amylose content for various countries of South and South East Asia

**Supplemental Table S5** Classification of improved lines based on grain shape, size and amylose content

**Supplemental Table S6** QTL/gene information data of developed improved lines forwarded further for multi‐location testing

**Supplemental Figure S1** Performance of improved lines under a). non‐stress and b). reproductive‐stage drought conditions
**Supplemental Figure S2** Blast resistant improved lines compared to susceptible checks
**Supplemental Figure S3** Gall midge a). improved resistant lines and b). susceptible checks

## Data Availability

The data sets supporting the results of this article are included within the article.
