# Supplementary material for: Genomics‐assisted breeding for successful development of multiple‐stress‐tolerant, climate‐smart rice for southern and southeastern Asia
Source: Plant Genome. 2021 Jan 12;14(1):e20074. doi: 10.1002/tpg2.20074 (PMC12806987; doi:10.1002/tpg2.20074)
Supplement: Supplementary file 1 [file TPG2-14-e20074-s003.pdf]

Full title

Genomics -assisted breeding enables successful development of multiple stress tolerant climate smart rice for South and South East Asia

Shailesh Yadav[1], Nitika Sandhu[1, 2], Shalabh Dixit[1], Vikas Kumar Singh[3], Margaret Catolos[1], Ratna Rani Mazumder[1,4], Mohammad Akhlasur Rahman[4] and Arvind Kumar[1,5*]

[1] Rice Breeding Platform, International Rice Research Institute, DAPO Box 7777, Metro Manila, Philippines

[2] Punjab Agricultural University, Ludhiana, Punjab, India

[3] International Rice Research Institute, South Asia Hub, ICRISAT, Patancheru, Hyderabad, India

[4] Bangladesh Rice Research Institute (BRRI), Gazipur, Bangladesh

[5] IRRI South Asia Regional Centre (ISARC), Varanasi, Uttar Pradesh, 221106, India

[*]Author for Correspondence

Arvind Kumar

International Rice Research Institute (IRRI)

IRRI South Asia Regional Centre (ISARC)

Varanasi-221006, Uttar Pradesh, India

Telephone: +91-542-2518900

Email: a.kumar@irri.org

ORICID ID: 0000-0002-5488-9410

SUPPORTING INFORMATION

Supplementary Tables (files are attached)

Table S1 Disease Reaction of improved lines against BLB and Blast disease

Table S2 Gall midge resistant improved lines

Table S3 Grain quality data of improved lines for product profiling to deploy in targeted breeding

Table S4 Classification of improved lines based on desired amylose content for various countries of South and South East Asia

Table S5 Classification of improved lines based on grain shape, size and amylose content

Table S6 QTLs/gene information data of developed improved lines forwarded further for multi-location testing

Supplementary Figures

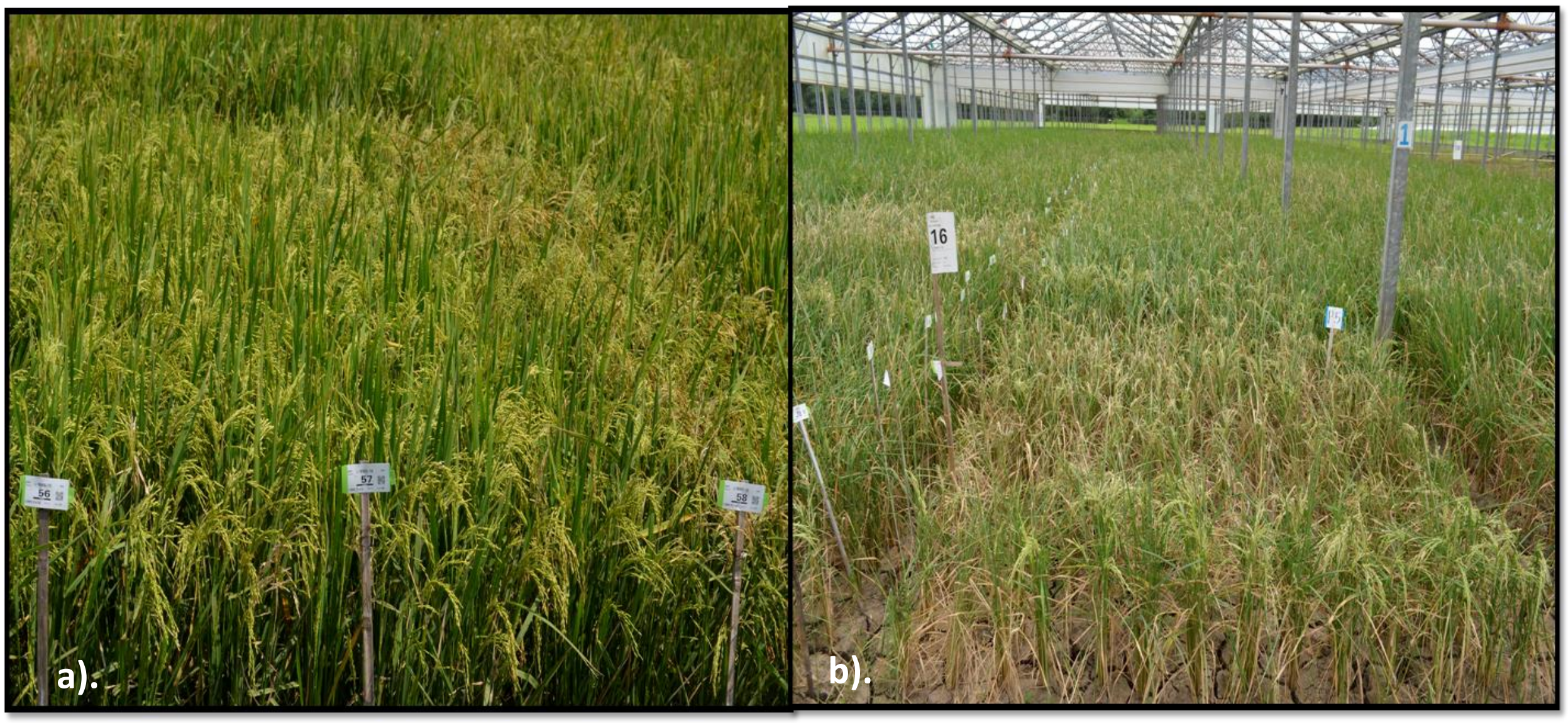

Figure S1 Performance of improved lines under a). non-stress and b). reproductive stage drought conditions

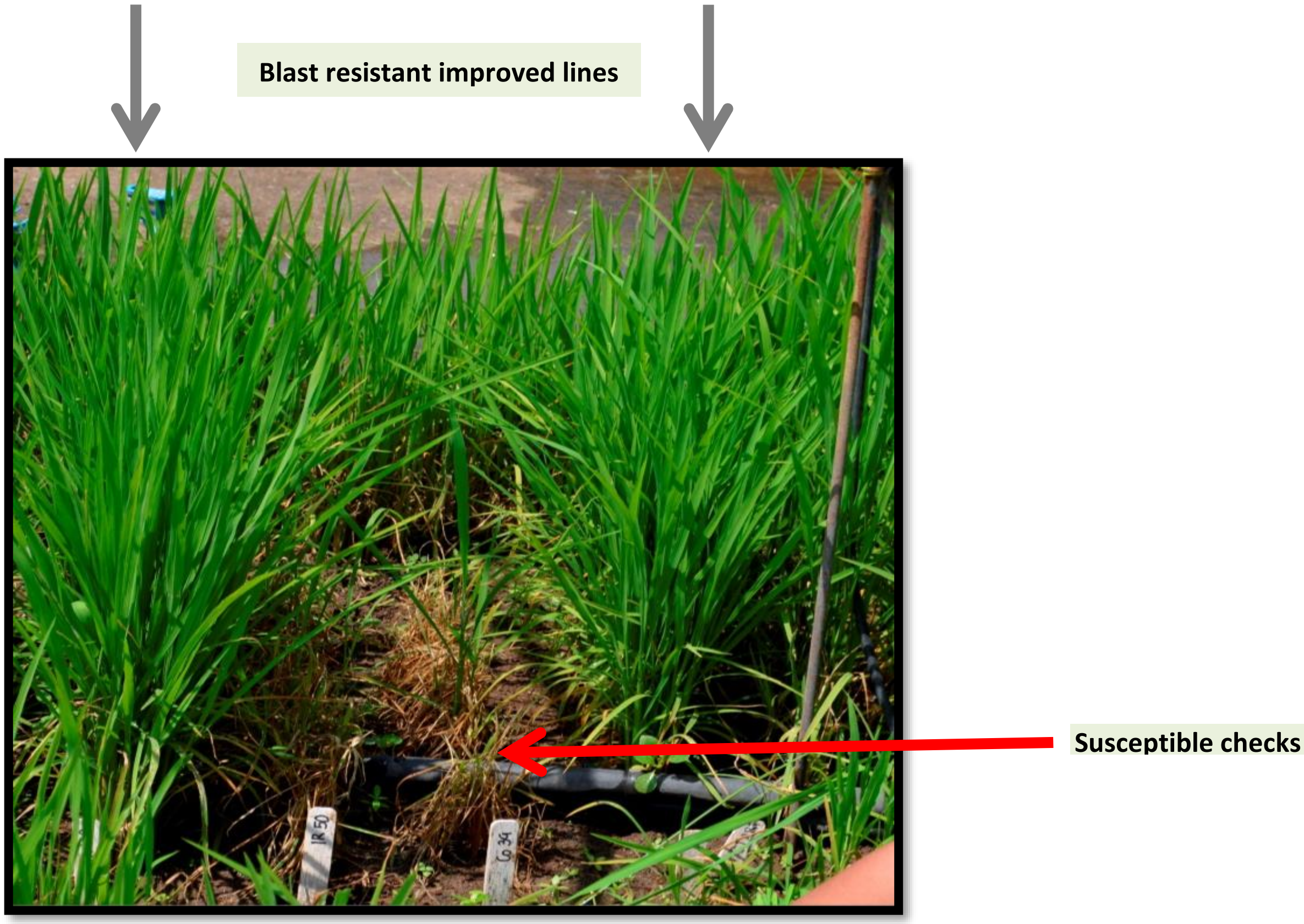

Figure S2 Blast resistant improved lines compared to susceptible checks

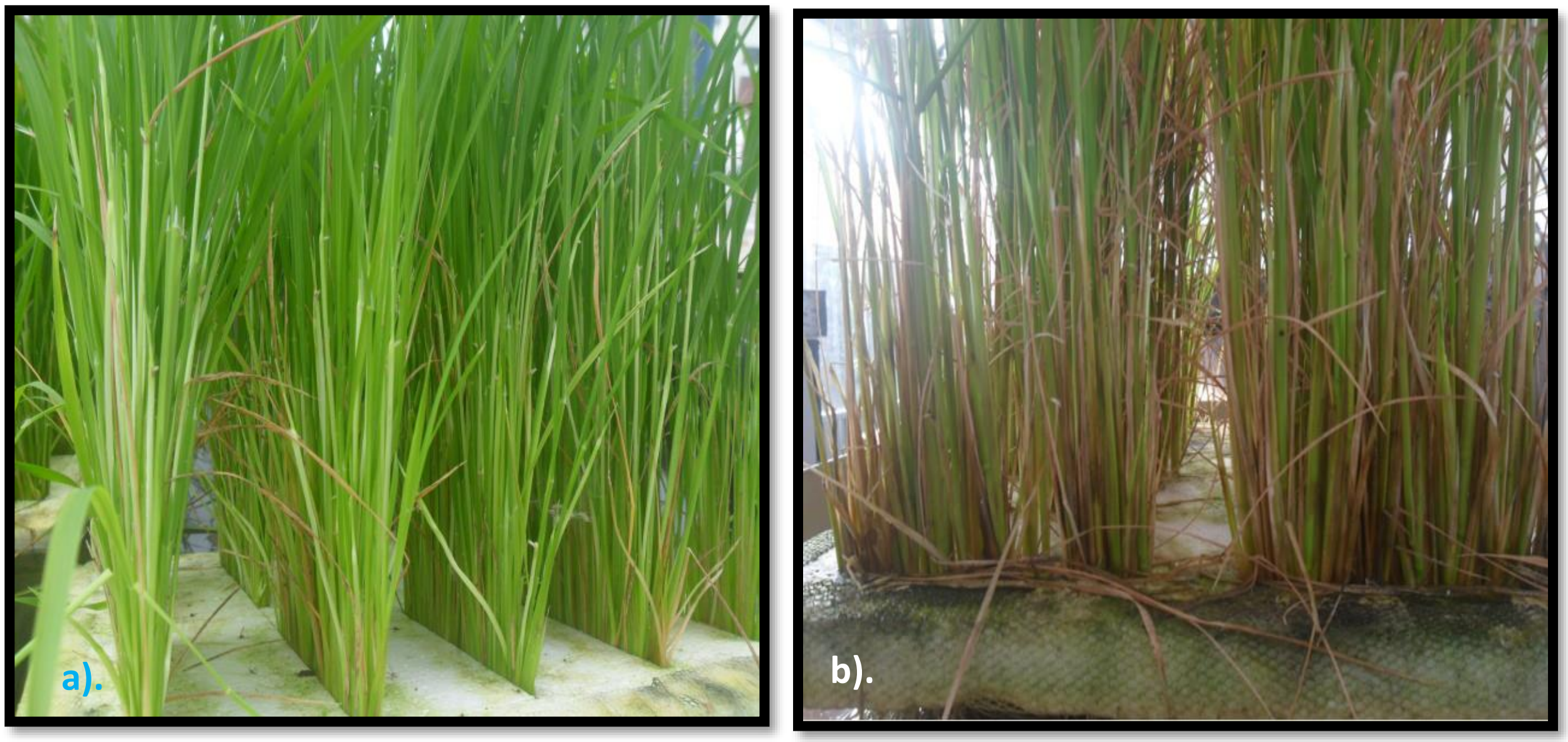

Figure S3 Gall midge a). improved resistant lines and b). susceptible checks
