# Supplementary material for: Genomics‐assisted breeding for successful development of multiple‐stress‐tolerant, climate‐smart rice for southern and southeastern Asia
Source: Plant Genome. 2021 Jan 12;14(1):e20074. doi: 10.1002/tpg2.20074 (PMC12806987; doi:10.1002/tpg2.20074)
Supplement: Supplementary file 2 [file TPG2-14-e20074-s001.docx]

**Table S1** Disease reaction of improved lines against BLB and Blast disease

| **Sl** | **Designation** | **BLB_PXO 61** | **BLB_PXO 86** | **Blast** |
| --- | --- | --- | --- | --- |
| 1 | IR18L1010 | 1 | 3 | 0 |
| 2 | IR18L1011 | 1 | 3 | 0 |
| 3 | IR18L1012 | 1 | 1 | 0 |
| 4 | IR18L1013 | 3 | 3 | 0 |
| 5 | IR18L1014 | 1 | 3 | 1 |
| 6 | IR18L1015 | 1 | 1 | 2 |
| 7 | IR18L1016 | 1 | 3 | 1 |
| 8 | IR18L1017 | 3 | 3 | 1 |
| 9 | IR18L1018 | 1 | 1 | 0 |
| 10 | IR18L1019 | 5 | 7 | 0 |
| 11 | IR18L1020 | 5 | 5 | 0 |
| 12 | IR18L1021 | 7 | 7 | 0 |
| 13 | IR18L1022 | 5 | 5 | 0 |
| 14 | IR18L1023 | 5 | 7 | 0 |
| 15 | IR18L1024 | 1 | 1 | 1 |
| 16 | IR18L1025 | 1 | 1 | 0 |
| 17 | IR18L1026 | 1 | 1 | 1 |
| 18 | IR18L1027 | 3 | 3 | 0 |
| 19 | IR18L1028 | 1 | 1 | 0 |
| 20 | IR18L1029 | 3 | 3 | 0 |
| 21 | IR18L1030 | 1 | 1 | 0 |
| 22 | IR18L1031 | 3 | 3 | 0 |
| 23 | IR18L1032 | 1 | 1 | 0 |
| 24 | IR18L1033 | 1 | 1 | 0 |
| 25 | IR18L1034 | 1 | 1 | 0 |
| 26 | IR18L1035 | 3 | 3 | 0 |
| 27 | IR18L1036 | 1 | 1 | 0 |
| 28 | IR18L1037 | 3 | 3 | 0 |
| 29 | IR18L1038 | 1 | 1 | 0 |
| 30 | IR18L1039 | 1 | 1 | 0 |
| 31 | IR18L1040 | 1 | 1 | 0 |
| 32 | IR18L1041 | 3 | 3 | 0 |
| 33 | IR18L1042 | 1 | 3 | 0 |
| 34 | IR18L1043 | 1 | 3 | 0 |
| 35 | IR18L1044 | 1 | 3 | 0 |
| 36 | IR18L1045 | 1 | 1 | 0 |
| 37 | IR18L1046 | 1 | 1 | 1 |
| 38 | IR18L1047 | 1 | 1 | 0 |
| 39 | IR18L1048 | 3 | 3 | 1 |
| 40 | IR18L1049 | 1 | 1 | 0 |
| 41 | IR18L1050 | 1 | 1 | 3 |
| 42 | IR18L1051 | 1 | 1 | 3 |
| 43 | IR18L1052 | 1 | 1 | 0 |
| 44 | IR18L1053 | 3 | 3 | 0 |
| 45 | IR18L1054 | 3 | 3 | 3 |
| 46 | IR18L1055 | 1 | 1 | 0 |
| 47 | IR18L1056 | 1 | 1 | 0 |
| 48 | IR18L1057 | 1 | 1 | 0 |
| 49 | IR18L1058 | 1 | 3 | 0 |
| 50 | IR18L1059 | 1 | 1 | 1 |
| 51 | IR18L1060 | 1 | 1 | 0 |
| 52 | IR18L1061 | 1 | 1 | 3 |
| 53 | IR18L1062 | 3 | 3 | 5 |
| 54 | IR18L1063 | 1 | 1 | 0 |
| 55 | IR18L1064 | 1 | 1 | 0 |
| 56 | IR18L1065 | 1 | 1 | 0 |
| 57 | IR18L1066 | 1 | 3 | 0 |
| 58 | IR18L1067 | 1 | 1 | 0 |
| 59 | IR18L1068 | 1 | 1 | 0 |
| 60 | IR18L1069 | 1 | 3 | 0 |
| 61 | IR18L1070 | 1 | 1 | 0 |
| 62 | IR18L1071 | 1 | 1 | 0 |
| 63 | IR18L1072 | 1 | 1 | 0 |
| 64 | IR18L1073 | 1 | 1 | 0 |
| 65 | IR18L1074 | 1 | 1 | 0 |
| 66 | IR18L1075 | 1 | 1 | 0 |
| 67 | IR18L1076 | 1 | 1 | 1 |
| 68 | IR18L1077 | 1 | 1 | 2 |
| 69 | IR18L1078 | 1 | 1 | 0 |
| 70 | IR18L1079 | 1 | 1 | 0 |
| 71 | IR18L1080 | 1 | 1 | 0 |
| 72 | IR18L1081 | 1 | 1 | 0 |
| 73 | IR18L1082 | 1 | 1 | 0 |
| 74 | IR18L1083 | 1 | 1 | 0 |
| 75 | IR18L1084 | 1 | 1 | 0 |
| 76 | IR18L1085 | 1 | 1 | 5 |
| 77 | IR18L1086 | 3 | 3 | 0 |
| 78 | IR18L1087 | 1 | 1 | 0 |
| 79 | IR18L1088 | 1 | 1 | 0 |
| 80 | IR18L1089 | 1 | 1 | 0 |
| 81 | IR18L1090 | 1 | 1 | 0 |
| 82 | IR18L1091 | 1 | 1 | 0 |
| 83 | IR18L1092 | 1 | 1 | 5 |
| 84 | IR18L1093 | 1 | 1 | 2 |
| 85 | IR18L1094 | 1 | 1 | 0 |
| 86 | IR18L1095 | 3 | 3 | 1 |
| 87 | IR18L1096 | 1 | 1 | 1 |
| 88 | IR18L1097 | 1 | 1 | 0 |
| 89 | IR18L1098 | 1 | 1 | 0 |
| 90 | IR18L1099 | 1 | 1 | 0 |
| 91 | IR18L1100 | 1 | 1 | 0 |
| 92 | IR18L1101 | 1 | 1 | 0 |
| 93 | IR18L1102 | 1 | 1 | 0 |
| 94 | IR18L1103 | 1 | 1 | 0 |
| 95 | IR18L1104 | 1 | 1 | 0 |
| 96 | IR 91648-B-1-B-3-1 | 7 | 7 | 9 |
