# Supplementary material for: Genomics‐assisted breeding for successful development of multiple‐stress‐tolerant, climate‐smart rice for southern and southeastern Asia
Source: Plant Genome. 2021 Jan 12;14(1):e20074. doi: 10.1002/tpg2.20074 (PMC12806987; doi:10.1002/tpg2.20074)
Supplement: Supplementary file 3 [file TPG2-14-e20074-s002.docx]

| **Designation** | **DTF-NS** | **PHT-NS** | **GY-NS** | **GY-RS** | **QTLs /gene combinations** | **No. of QTLs/genes** | **BLB**  **(SES Score)** | **Blast**  **(SES Score** | **Chalkiness**  **(%)** | **AC**  **(%)** | **GT** | **% HRR** | **Targeted countries** |
| --- | --- | --- | --- | --- | --- | --- | --- | --- | --- | --- | --- | --- | --- |
| IR18L1149 | 86 | 102 | 7427 | 1777 | *qDTY_1.1_+qDTY_2.1_+qDTY_3.1_+Sub1+Pi9+Xa21+Xa23* | 7 | 1 | 0 | 6.53 | 25.1 | I | 53.3 | Bangladesh, Sri -Lanka, Myanmar, |
| IR18L1129 | 87 | 102 | 8043 | 1474 | *qDTY_1.1_+qDTY_3.1_+qDTY_12.1_+Sub1+Gm4+ Pi9+xa5+Xa21+Xa23* | 9 | 1 | 1 | 5.31 | 24.7 | I | 62.2 | India, Philippines, Nepal |
| IR18L1147 | 87 | 104 | 7470 | 1581 | *qDTY_1.1_+qDTY_2.1_+qDTY_12.1_+Sub1+Gm4+ Pi9+xa5+Xa21+Xa23+Bph17* | 10 | 1 | 2 | 1.27 | 24.6 | I | 66.8 | India, Philippines, Nepal |
| IR18L1151 | 88 | 112 | 8146 | 2300 | *qDTY_1.1_+qDTY_3.1_+qDTY_12.1_+Sub1+Gm4+ Pi9+xa5+Xa21+Xa23* | 9 | 1 | 0 | 5.68 | 24.6 | I | 58.5 | India, Philippines, Nepal |
| IR18L1130 | 89 | 102 | 7571 | 1721 | *qDTY_1.1_+qDTY_3.1_+qDTY_12.1_+Sub1+Gm4+ Pi9+xa5+Xa21+Xa23+Bph17* | 10 | 1 | 1 | 4.07 | 24.4 | I | 65.8 | India, Philippines, Nepal |
| IR18L1127 | 87 | 109 | 7086 | 1425 | *qDTY_1.1_+qDTY_2.1_+qDTY_3.1_+qDTY_12.1_+Sub1+Gm4+Pi9+xa5+Xa21+Bph17* | 10 | 1 | 1 | 1.86 | 21.4 | I | 61.3 | India, Philippines, Nepal |
| IR18L1156 | 83 | 91 | 7988 | 2002 | *qDTY_1.1_+qDTY_12.1_+Sub1+Pi9+Gm4+Xa4+xa5+Xa21+Xa23+Bph3* | 10 | 1 | 0 | 4.89 | 21.3 | HI | 61.4 | India, Philippines, Nepal |
| IR18L1165 | 81 | 117 | 7565 | 1446 | *qDTY_1.1_+qDTY_3.1_+qDTY_12.1_+Sub1+Pi9+xa5+Xa21+Xa23+Bph3* | 9 | 1 | 0 | 5.45 | 20.5 | I | 61 | India, Philippines, Nepal |
| IR18L1157 | 83 | 94 | 7084 | 976 | *qDTY_1.1_+qDTY_12.1_+Sub1+Pi9+Gm4+xa5+Xa21+Xa23+Bph3* | 9 | 1 | 1 | 2.7 | 20.4 | HI | 63 | India, Philippines, Nepal |
| IR18L1161 | 86 | 97 | 6823 | 1894 | *qDTY_1.1_+Sub1+Pi9+GM4+xa5+Xa21+Xa23+Bph3+Bph17* | 9 | 1 | 0 | 1.84 | 20.4 | I | 65.4 | India, Philippines, Nepal |
| IR18L1163 | 82 | 94 | 7293 | 1683 | *qDTY_1.1_+Sub1+Pi9+GM4+xa5+Xa21+Xa23+Bph3+Bph17* | 9 | 1 | 0 | 1.15 | 20.4 | I | 64.1 | India, Philippines, Nepal |
| IR18L1143 | 83 | 101 | 7307 | 1677 | *qDTY_1.1_+qDTY_12.1_+Sub1+Pi9+Gm4+xa5+Xa21+Xa23+Bph3+Bph17* | 10 | 3 | 1 | 3 | 20.2 | I | 55 | India, Philippines, Nepal |
| IR18L1132 | 87 | 100 | 6188 | 1474 | *qDTY_1.1_+qDTY_12.1_+Sub1+Pi9+xa5+Xa21+Xa23+Bph3* | 8 | 1 | 0 | 5.29 | 19.8 | HI | 53 | LaoPDR, Cambodia, Japan, Taiwan |
| IR18L1152 | 88 | 107 | 7684 | 1532 | *qDTY_1.1_+Sub1+Pi9+Gm4+xa5+Xa21+Xa23+Bph17* | 8 | 1 | 0 | 1.86 | 19.6 | HI | 61.3 | LaoPDR, Cambodia, Japan, Taiwan |
| IR18L1154 | 85 | 95 | 7837 | 1770 | *qDTY_1.1_+Sub1+Pi9+Gm4+xa5+Xa23+Bph3+Bph17* | 8 | 1 | 0 | 1.86 | 19.6 | HI | 61.3 | LaoPDR, Cambodia, Japan, Taiwan |
| IR18L1142 | 86 | 100 | 6511 | 1636 | *qDTY_1.1_+qDTY_2.1_+qDTY_12.1_+Sub1+Pi9+Gm4+xa5+Bph3+Bph17* | 9 | 1 | 0 | 4.68 | 19.3 | HI | 48.5 | LaoPDR, Cambodia, Japan, Taiwan, |
| IR18L1148 | 84 | 90 | 6869 | 1581 | *qDTY_3.1_+qDTY_12.1_+Sub1+Pi9+Gm4+Bph3+Bph17* | 7 | 5 | 0 | 4.25 | 16.9 | HI | 52.3 | LaoPDR, Cambodia, Japan, Taiwan |

**Supplementary Table S4:** Classification of improved lines based on desired amylose content for various countries of South and South East Asia

**Note:** DTF-NS(days to 50% flowering under non stress); PHT-NS (plant height under non stress);GY-NS (grain yield under non stress);

GY-RS(grain yield under reproductive stage drought stress); BLB (bacterial leaf blight); AC (amylose content); GT(gelatinization temperature); I-Intermediate GT ;

HI-high GT; %HRR(head rice recovery in percentage) ; BLB and Blast score: 0- immune; 1-highly resistant; 3- resistant ; 5- moderately resistant ;7-susceptible;

9- highly susceptible(IRRI SES, 2002)
