# Supplementary material for: Genomics‐assisted breeding for successful development of multiple‐stress‐tolerant, climate‐smart rice for southern and southeastern Asia
Source: Plant Genome. 2021 Jan 12;14(1):e20074. doi: 10.1002/tpg2.20074 (PMC12806987; doi:10.1002/tpg2.20074)
Supplement: Supplementary file 4 [file TPG2-14-e20074-s004.docx]

**Table S6** QTLs/gene information data of developed improved lines forwarded further for multi-location testing

| **Designation** | **PH_NS** | **DTF_NS** | **GYKGPHA_NS** | **No. of genes** | **Chalkiness** | **AC** | **GT** | **% HRR** | **QTLs /genes** |
| --- | --- | --- | --- | --- | --- | --- | --- | --- | --- |
| IR18L1127 | 109 | 87 | 7086 | 10 | 1.86 | 21.4 | I | 61.3 | *qDTY_1.1_+qDTY_2.1_+qDTY_3.1_+qDTY_12.1_+Sub1+GM4+Pi9+xa5+Xa21+BPH17* |
| IR18L1128 | 109 | 90 | 7440 | 7 | 5.31 | 24.7 | I | 62.2 | *qDTY_1.1_+qDTY_3.1_+Sub1+GM4+xa5+Xa23+BPH3* |
| IR18L1129 | 102 | 87 | 8043 | 9 | 5.31 | 24.7 | I | 62.2 | *qDTY_1.1_+qDTY_3.1_+qDTY_12.1_+Sub1+GM4+Pi9+xa5+Xa21+Xa23* |
| IR18L1130 | 102 | 89 | 7571 | 10 | 2.01 | 24.1 | I | 64.6 | *qDTY_1.1_+qDTY_3.1_+qDTY_12.1_+Sub1+GM4+ Pi9+xa5+Xa21+Xa23+BPH17* |
| IR18L1131 | 111 | 91 | 7866 | 10 | 4.07 | 24.4 | I | 65.8 | *qDTY_1.1_+qDTY_3.1_+qDTY_12.1_+Sub1+GM4+Xa4+xa5+Xa21+Xa23+BPH17* |
| IR18L1132 | 100 | 87 | 6188 | 9 | 5.29 | 19.8 | HI | 53 | *qDTY_1.1_+qDTY_12.1_+Sub1+Pi9+xa5+Xa21+Xa23+BPH3* |
| IR18L1133 | 107 | 71 | 6512 | 7 | 22.76 | 23.4 | I | 52.6 | *qDTY_1.1_+qDTY_2.1_+qDTY_3.1_+Sub1+xa5+xa13+Pita2* |
| IR18L1134 | 94 | 93 | 6842 | 8 | 1.73 | 24.8 | I | 53.1 | *qDTY_1.1_+qDTY_2.1_+qDTY_12.1_+Sub1+Pi9+GM4+xa5+BPH17* |
| IR18L1135 | 103 | 88 | 6749 | 7 | 18.62 | 22.9 | HI | 30.6 | *qDTY_1.1_+qDTY_2.1_+qDTY_3.1_+qDTY_12.1_+Pi9+BPH3+BPH17* |
| IR18L1136 | 113 | 91 | 8050 | 10 | 0.78 | 24.5 | I | 63.1 | *qDTY_1.1_+qDTY_2.1_+qDTY_12.1_+Sub1+GM4+Xa4+xa5+Xa21+Xa23+BPH17* |
| IR18L1137 | 107 | 89 | 7964 | 10 | 1.86 | 21.4 | I | 61.3 | *qDTY_1.1_+qDTY_2.1_+qDTY_3.1_+qDTY_12.1_+Sub1+GM4+Xa4+Xa21+Xa23+BPH17* |
| IR18L1138 | 115 | 84 | 8020 | 9 | 5.45 | 20.5 | HI/I | 61 | *qDTY_1.1_+qDTY_3.1_+qDTY_12.1_+Sub1+Pi9+xa5+Xa21+Xa23+BPH3* |
| IR18L1139 | 113 | 88 | 8389 | 6 | 16.25 | 24.8 | I | 37 | *qDTY_3.1_+Sub1+GM4+xa5+xa13+Pita2* |
| IR18L1140 | 108 | 83 | 9586 | 6 | 6.22 | 20.3 | HI/I | 61.6 | *qDTY_1.1_+qDTY_2.1_+qDTY_3.1_+qDTY_12.1_+Sub1+xa5* |
| IR18L1141 | 114 | 95 | 8310 | 8 | 4.86 | 20.7 | I | 57.6 | *qDTY_1.1_+qDTY_2.1_+qDTY_3.1_+Pi9+GM4+xa5+BPH3+BPH17* |
| IR18L1142 | 100 | 86 | 6511 | 9 | 4.68 | 19.3 | HI | 48.5 | *qDTY_1.1_+qDTY_2.1_+qDTY_12.1_+Sub1+Pi9+GM4+xa5+BPH3+BPH17* |
| IR18L1143 | 101 | 83 | 7307 | 10 | 2.9 | 20.0 | I | 55.6 | *qDTY_1.1_+qDTY_12.1_+Sub1+Pi9+GM4+xa5+Xa21+Xa23+BPH3+BPH17* |
| IR18L1144 | 111 | 90 | 6957 | 8 | 1.7 | 20.0 | HI/I | 44.8 | *qDTY_1.1_+qDTY_2.1_+qDTY_12.1_+Sub1+Pi9+GM4+BPH3+BPH17* |
| IR18L1145 | 107 | 86 | 7828 | 9 | 2.01 | 20.0 | HI | 46.9 | *qDTY_2.1_+qDTY_3.1_+qDTY_12.1_+Sub1+Pi9+GM4+xa5+BPH3+BPH17* |
| IR18L1146 | 109 | 90 | 5712 | 10 | 2.28 | 21.2 | I | 59.1 | *qDTY_1.1_+qDTY_12.1_+Sub1+Pi9+GM4+xa5+Xa21+Xa23+BPH3+BPH17* |
| IR18L1147 | 104 | 87 | 7470 | 10 | 1.27 | 24.6 | I/L | 66.8 | *qDTY_1.1_+qDTY_2.1_+qDTY_12.1_+Sub1+GM4+ Pi9+xa5+Xa21+Xa23+BPH17* |
| IR18L1148 | 90 | 84 | 6869 | 7 | 4.25 | 16.9 | HI/I | 52.3 | *qDTY_3.1_+qDTY_12.1_+Sub1+Pi9+GM4+BPH3+BPH17* |
| IR18L1149 | 102 | 86 | 7427 | 7 | 6.53 | 25.0 | I | 53.3 | *qDTY_1.1_+qDTY_2.1_+qDTY_3.1_+Sub1+Pi9+Xa21+Xa23* |
| IR18L1150 | 116 | 87 | 7241 | 7 | 6.44 | 20.6 | HI/I | 54.6 | *qDTY_1.1_+qDTY_2.1_+qDTY_3.1_+Sub1+ Pi9+Xa21+Xa23+BPH3* |
| IR18L1151 | 112 | 88 | 8146 | 9 | 5.68 | 24.6 | I | 58.5 | *qDTY_1.1_+qDTY_3.1_+qDTY_12.1_+Sub1+GM4+ Pi9+xa5+Xa21+Xa23* |
| IR18L1152 | 107 | 88 | 7684 | 8 | 1.86 | 19.6 | HI/I | 61.3 | *qDTY_1.1_+Sub1+Pi9+GM4+xa5+Xa21+Xa23+BPH17* |
| IR18L1153 | 107 | 89 | 6899 | 8 | 15.54 | 20.5 | HI | 31.4 | *qDTY_1.1_+qDTY_3.1_+qDTY_12.1_+Sub1+xa5+Xa21+BPH3+BPH17* |
| IR18L1154 | 95 | 85 | 7837 | 8 | 1.86 | 19.6 | HI/I | 61.3 | *qDTY_1.1_+Sub1+Pi9+GM4+xa5+Xa23+BPH3+BPH17* |
| IR18L1155 | 108 | 88 | 5582 | 9 | 0.83 | 21.1 | HI | 61.9 | *qDTY_1.1_++qDTY_2.1_+qDTY_12.1_+Sub1+Pi9+GM4+xa5+Xa21+Xa23* |
| IR18L1156 | 91 | 83 | 7988 | 10 | 4.89 | 21.3 | HI | 61.4 | *qDTY_1.1_+qDTY_12.1_+Sub1+Pi9+GM4+Xa4+xa5+Xa21+Xa23+BPH3* |
| IR18L1157 | 94 | 83 | 7084 | 9 | 2.7 | 20.4 | HI | 63 | *qDTY_1.1_+qDTY_12.1_+Sub1+Pi9+GM4+xa5+Xa21+Xa23+BPH3* |
| IR18L1158 | 103 | 91 | 7423 | 8 | 3.48 | 20.6 | HI/I | 61.2 | *qDTY_1.1_+Sub1+Pi9+GM4+xa5+Xa21+Xa23+BPH17* |
| IR18L1159 | 91 | 83 | 6158 | 10 | 5.89 | 20.7 | I | 47.2 | *qDTY_1.1_+qDTY_2.1_+qDTY_3.1_+qDTY_12.1_+Sub1+Pi9+GM4+xa5+BPH3+BPH17* |
| IR18L1160 | 118 | 84 | 7716 | 9 | 5.45 | 20.5 | HI/I | 61 | *qDTY_1.1_+qDTY_3.1_+qDTY_12.1_+Sub1+Pi9+xa5+Xa21+Xa23+BPH3* |
| IR18L1161 | 97 | 86 | 6823 | 9 | 1.84 | 20.4 | I | 65.4 | *qDTY_1.1_+Sub1+Pi9+GM4+xa5+Xa21+Xa23+BPH3+BPH17* |
| IR18L1162 | 107 | 86 | 7839 | 7 | 5.34 | 20.1 | HI/I | 52.2 | *qDTY_1.1_+qDTY_2.1_+qDTY_3.1_+Xa4+xa5+BPH3+BPH17* |
| IR18L1163 | 94 | 82 | 7293 | 9 | 1.15 | 20.4 | I | 64.1 | *qDTY_1.1_+Sub1+Pi9+GM4+xa5+Xa21+Xa23+BPH3+BPH17* |
| IR18L1164 | 88 | 81 | 5775 | 10 | 4.77 | 23.7 | I | 53.4 | *qDTY_1.1_+qDTY_2.1_+qDTY_3.1_+qDTY_12.1_+Sub1+Pi9+GM4+xa5+BPH3+BPH17* |
| IR18L1165 | 117 | 81 | 7565 | 9 | 5.45 | 20.5 | HI/I | 61 | *qDTY_1.1_+qDTY_3.1_+qDTY_12.1_+Sub1+Pi9+xa5+Xa21+Xa23+BPH3* |
| IR18L1166 | 93 | 82 | 8551 | 9 | 5.11 | 23.7 | I | 33.4 | *qDTY_1.1_+qDTY_2.1_+qDTY_3.1_+qDTY_12.1_+Sub1+Pi9+GM4+xa5+BPH17* |
| IR18L1167 | 90 | 82 | 6924 | 9 | 3.48 | 23.5 | I | 57 | *qDTY_2.1_+qDTY_3.1_+qDTY_12.1_+Sub1+Pi9+GM4+xa5+BPH3+BPH17* |
| IR18L1168 | 110 | 83 | 7769 | 8 | 0.85 | 20.6 | HI/I | 61.2 | *qDTY_1.1_+qDTY_12.1_+Sub1+Pi9+GM4+xa5+Xa21+Xa23* |
| IR18L1169 | 103 | 81 | 7627 | 6 | 2.43 | 19.5 | HI | 60.3 | *qDTY_1.1_+qDTY_2.1_+Sub1+Pi9+xa5+Xa23* |
| IR18L1170 | 103 | 81 | 8445 | 8 | 5.77 | 20.5 | HI/I | 55.2 | *qDTY_3.1_+qDTY_12.1_+Sub1+Pi9+xa5+Xa21+Xa23+BPH3* |
| IR18L1171 | 93 | 82 | 6874 | 9 | 1.88 | 20.2 | I | 53.3 | *qDTY_1.1_+qDTY_2.1_+qDTY_3.1_+qDTY_12.1_+Sub1+Pi9+GM4+xa5+BPH17* |
| IR18L1172 | 96 | 84 | 6076 | 9 | 1.71 | 20.4 | HI | 53.6 | *qDTY_1.1_+qDTY_2.1_+qDTY_3.1_+qDTY_12.1_+Sub1+Pi9+GM4+xa5+BPH17* |
| IR18L1173 | 104 | 83 | 7158 | 9 | 5.61 | 20.3 | HI | 56.6 | *qDTY_1.1_+qDTY_2.1_+qDTY_3.1_+Sub1+Pi9+Xa21+Xa23+BPH3+BPH17* |
| IR18L1174 | 117 | 89 | 6683 | 8 | 5.29 | 20.3 | I | 53 | *qDTY_1.1_+qDTY_2.1_+qDTY_3.1_+qDTY_12.1_+Pi9+GM4+BPH3+BPH17* |
| IR18L1175 | 113 | 90 | 6060 | 8 | 2.96 | 20.3 | HI | 59.4 | *qDTY_1.1_+qDTY_2.1_+qDTY_3.1_+qDTY_12.1_+Pi9+GM4+Xa23+BPH17* |
| IR18L1176 | 97 | 86 | 7196 | 7 | 2.92 | 21.4 | HI | 51.8 | *qDTY_3.1_+qDTY_12.1_+Sub1+Pi9 +xa5+Xa21+Xa23* |
| IR18L1177 | 100 | 85 | 6067 | 8 | 1.86 | 20.3 | HI/I | 61.3 | *qDTY_3.1_+qDTY_12.1_+Sub1+Pi9+xa5+Xa21+Xa23+BPH3* |
| IR18L1178 | 110 | 84 | 8096 | 8 | 1.72 | 20.2 | HI/I | 56.9 | *qDTY_3.1_+qDTY_12.1_+Sub1+Pi9+ xa5+Xa21+Xa23+BPH3* |
| IR18L1179 | 102 | 83 | 8154 | 8 | 5.95 | 20.1 | I | 54.8 | *qDTY_3.1_+qDTY_12.1_+Sub1+Pi9+xa5+Xa21+Xa23+BPH3* |
| IR18L1180 | 104 | 86 | 8235 | 8 | 3.4 | 21.4 | I | 55.4 | *qDTY_3.1_+qDTY_12.1_+Sub1+Pi9+xa5+Xa21+Xa23+BPH3* |
| IR18L1181 | 102 | 87 | 6799 | 9 | 2.04 | 20.2 | HI | 43.9 | *qDTY_1.1_+Sub1+Pi9+GM4+xa5+Xa21+Xa23+BPH3+BPH17* |
| IR18L1182 | 112 | 87 | 7534 | 10 | 3 | 20.0 | I | 55 | *qDTY_1.1_+qDTY_12.1_+Sub1+Pi9+GM4+xa5+Xa21+Xa23+BPH3+BPH17* |
| IR 91648-B-1-B-3-1 | 101 | 82 | 7351 |  | 1.55 | 21.3 | HI | 59 |  |
| IR64 | 107 | 80 | 6974 |  | 9.37 | 19.7 | HI | 51.8 |  |
| MTU 1010 | 91 | 81 | 7413 |  | 4.79 | 20.0 | HI/I | 64.5 |  |
| Swarna | 75 | 102 |  |  | 0.78 | 25.2 | I | 45.8 |  |
| Mean | 103 | 86 | 7318 |  |  |  |  |  |  |
| SED | 4 | 2 | 550 |  |  |  |  |  |  |
| LSD | 8 | 4 | 1100 |  |  |  |  |  |  |
| H | 0.78 | 0.92 | 0.82 |  |  |  |  |  |  |
