# Supplementary material for: Genomics‐assisted breeding for successful development of multiple‐stress‐tolerant, climate‐smart rice for southern and southeastern Asia
Source: Plant Genome. 2021 Jan 12;14(1):e20074. doi: 10.1002/tpg2.20074 (PMC12806987; doi:10.1002/tpg2.20074)
Supplement: Supplementary file 5 [file TPG2-14-e20074-s005.docx]

**Table S5 Classification of improved lines based on grain shape, size and amylose content**

| **Sl** | **Designation** | **GID** | **Grain length** | **Length** | **Grain width** | **L-B Ratio** | **Grain shape** | **Gelatinization temp** | **% Amylose** | **Amylose category** |
| --- | --- | --- | --- | --- | --- | --- | --- | --- | --- | --- |
| 1 | IR18L1010 | 300986335 | 7.02 | Long | 2.3 | 3.1 | Slender | I | 26.4 | H |
| 2 | IR18L1011 | 300986292 | 6.95 | Long | 2.3 | 3.0 | Medium | I | 25.6 | H |
| 3 | IR18L1012 | 300986293 | 7.00 | Long | 2.29 | 3.1 | Slender | I | 25.1 | H |
| 4 | IR18L1013 | 300986294 | 7.1 | Long | 2.33 | 3.0 | Medium | I | 25.1 | H |
| 5 | IR18L1014 | 300986295 | 6.36 | Medium | 2.42 | 2.6 | Medium | I | 24.8 | I |
| 6 | IR18L1015 | 300986296 | 6.32 | Medium | 2.43 | 2.6 | Medium | I | 24.8 | I |
| 7 | IR18L1016 | 300986297 | 6.33 | Medium | 2.36 | 2.7 | Medium | I | 24.7 | I |
| 8 | IR18L1017 | 300986298 | 6.35 | Medium | 2.37 | 2.7 | Medium | I | 24.7 | I |
| 9 | IR18L1018 | 300986299 | 6.22 | Medium | 2.38 | 2.6 | Medium | I/L | 24.6 | I |
| 10 | IR18L1019 | 300986300 | 6.62 | Long | 2.2 | 3.1 | Slender | I | 24.6 | I |
| 11 | IR18L1020 | 300986301 | 6.73 | Long | 2.03 | 3.3 | Slender | I | 24.5 | I |
| 12 | IR18L1021 | 300986302 | 6.59 | Medium | 2.19 | 3.1 | Slender | I | 24.4 | I |
| 13 | IR18L1022 | 300986303 | 6.67 | Long | 2.17 | 3.1 | Slender | I | 24.3 | I |
| 14 | IR18L1023 | 300986304 | 6.74 | Long | 2.21 | 3.0 | Medium | I | 24 | I |
| 15 | IR18L1024 | 300986305 | 6.16 | Medium | 2.43 | 2.5 | Medium | I | 24 | I |
| 16 | IR18L1025 | 300986306 | 6.25 | Medium | 2.36 | 2.6 | Medium | I | 23.7 | I |
| 17 | IR18L1026 | 300986314 | 6.24 | Medium | 2.38 | 2.6 | Medium | I | 23.7 | I |
| 18 | IR18L1027 | 300986307 | 6.76 | Long | 2.23 | 3.0 | Medium | I | 23.7 | I |
| 19 | IR18L1028 | 300986315 | 6.79 | Long | 2.26 | 3.1 | Slender | I | 23.6 | I |
| 20 | IR18L1029 | 300986316 | 6.83 | Long | 2.24 | 3.0 | Medium | I | 23.5 | I |
| 21 | IR18L1030 | 300986317 | 6.84 | Long | 2.26 | 3.0 | Slender | I | 23.4 | I |
| 22 | IR18L1031 | 300986308 | 6.82 | Long | 2.2 | 3.1 | Slender | I | 23.4 | I |
| 23 | IR18L1032 | 300986318 | 6.84 | Long | 2.2 | 3.1 | Slender | I | 23 | I |
| 24 | IR18L1033 | 300986319 | 6.78 | Long | 2.25 | 3.0 | Medium | I | 22.9 | I |
| 25 | IR18L1034 | 300986320 | 6.88 | Long | 2.2 | 3.1 | Slender | HI | 22.9 | I |
| 26 | IR18L1035 | 300986309 | 6.75 | Long | 2.19 | 3.1 | Slender | I | 22.8 | I |
| 27 | IR18L1036 | 300986322 | 6.65 | Long | 2.15 | 3.1 | Slender | I | 22.6 | I |
| 28 | IR18L1037 | 300986323 | 6.63 | Long | 2.17 | 3.4 | Slender | I | 22.3 | I |
| 29 | IR18L1038 | 300986324 | 6.82 | Long | 2.23 | 3.1 | Slender | I | 22.2 | I |
| 30 | IR18L1039 | 300986310 | 6.88 | Long | 2.32 | 3.0 | Medium | I | 22.2 | I |
| 31 | IR18L1040 | 300986325 | 6.80 | Long | 2.3 | 3.0 | Medium | I | 22.2 | I |
| 32 | IR18L1041 | 300986326 | 6.72 | Long | 2.18 | 3.1 | Slender | HI/I | 22.2 | I |
| 33 | IR18L1042 | 300986327 | 6.95 | Long | 2.15 | 3.2 | Slender | I | 22.2 | I |
| 34 | IR18L1043 | 300986311 | 6.94 | Long | 2.13 | 3.3 | Slender | I | 22.1 | I |
| 35 | IR18L1044 | 300986328 | 6.90 | Long | 2.29 | 3.0 | Medium | HI | 21.9 | I |
| 36 | IR18L1045 | 300986329 | 6.89 | Long | 2.3 | 3.1 | Slender | I | 21.9 | I |
| 37 | IR18L1046 | 300986336 | 6.46 | Medium | 2.4 | 2.7 | Medium | L | 21.8 | I |
| 38 | IR18L1047 | 300986330 | 6.94 | Long | 2.02 | 3.4 | Slender | HI/I | 21.7 | I |
| 39 | IR18L1048 | 300986331 | 6.81 | Long | 2.35 | 3.0 | Medium | I | 21.4 | I |
| 40 | IR18L1049 | 300986332 | 6.52 | Medium | 2.25 | 2.9 | Medium | I | 21.4 | I |
| 41 | IR18L1050 | 300986312 | 6.51 | Medium | 2.44 | 2.7 | Medium | I | 21.4 | I |
| 42 | IR18L1051 | 300986333 | 6.38 | Medium | 2.48 | 2.6 | Medium | I | 21.3 | I |
| 43 | IR18L1052 | 300986334 | 6.47 | Medium | 2.46 | 2.6 | Medium | I | 21.3 | I |
| 44 | IR18L1053 | 300986321 | 6.76 | Long | 2.21 | 3.2 | Slender | HI | 21.3 | I |
| 45 | IR18L1054 | 300986339 | 6.77 | Long | 2.21 | 3.5 | Slender | HI | 21.3 | I |
| 46 | IR18L1055 | 300986340 | 7.03 | Long | 2.15 | 3.3 | Slender | I | 21.2 | I |
| 47 | IR18L1056 | 300986341 | 7.04 | Long | 2.12 | 3.3 | Slender | HI | 21.2 | I |
| 48 | IR18L1057 | 300986338 | 7.05 | Long | 2.17 | 3.2 | Slender | HI | 21.1 | I |
| 49 | IR18L1058 | 300986342 | 6.83 | Long | 2.47 | 2.8 | Medium | HI | 21 | I |
| 50 | IR18L1059 | 300986343 | 6.72 | Long | 2.37 | 2.8 | Medium | HI/I | 20.9 | I |
| 51 | IR18L1060 | 300986344 | 6.80 | Long | 2.45 | 2.8 | Medium | I | 20.7 | I |
| 52 | IR18L1061 | 300986337 | 6.73 | Long | 2.44 | 2.8 | Medium | I | 20.7 | I |
| 53 | IR18L1062 | 300986345 | 6.73 | Long | 2.03 | 3.3 | Slender | HI/I | 20.6 | I |
| 54 | IR18L1063 | 300986346 | 6.87 | Long | 2.1 | 3.3 | Slender | HI | 20.6 | I |
| 55 | IR18L1064 | 300986347 | 6.81 | Long | 2.22 | 3.1 | Slender | HI/I | 20.6 | I |
| 56 | IR18L1065 | 300986313 | 6.53 | Medium | 2.17 | 3.1 | Slender | HI/I | 20.6 | I |
| 57 | IR18L1066 | 300986348 | 6.57 | Medium | 2.16 | 3.0 | Medium | HI/I | 20.6 | I |
| 58 | IR18L1067 | 300986349 | 7.00 | Long | 2.14 | 3.3 | Slender | HI/I | 20.5 | I |
| 59 | IR18L1068 | 300986371 | 6.86 | Long | 2.1 | 3.3 | Slender | HI/I | 20.5 | I |
| 60 | IR18L1069 | 300986350 | 7.06 | Long | 2.31 | 3.1 | Slender | HI | 20.5 | I |
| 61 | IR18L1070 | 300986351 | 7.19 | Long | 2.35 | 3.1 | Slender | I | 20.4 | I |
| 62 | IR18L1071 | 300986352 | 6.76 | Long | 2.07 | 3.3 | Slender | I | 20.4 | I |
| 63 | IR18L1072 | 300986353 | 6.75 | Long | 2.24 | 3.0 | Medium | HI | 20.4 | I |
| 64 | IR18L1073 | 300986354 | 6.78 | Long | 2.41 | 2.8 | Medium | HI | 20.4 | I |
| 65 | IR18L1074 | 300986355 | 6.69 | Long | 2.4 | 2.8 | Medium | I | 20.3 | I |
| 66 | IR18L1075 | 300986373 | 6.71 | Long | 2.41 | 2.8 | Medium | HI | 20.3 | I |
| 67 | IR18L1076 | 300986356 | 5.90 | Medium | 2.48 | 2.4 | Medium | HI/I | 20.3 | I |
| 68 | IR18L1077 | 300986357 | 6.14 | Medium | 2.47 | 2.5 | Medium | HI/I | 20.2 | I |
| 69 | IR18L1078 | 300986358 | 6.85 | Long | 2.09 | 3.3 | Slender | I | 20.1 | I |
| 70 | IR18L1079 | 300986359 | 6.98 | Long | 2.31 | 3.0 | Medium | HI/I | 20.1 | I |
| 71 | IR18L1080 | 300986360 | 6.76 | Long | 2.12 | 3.2 | Slender | HI | 20.1 | I |
| 72 | IR18L1081 | 300986361 | 6.78 | Long | 2.16 | 3.1 | Slender | HI/I | 20.1 | I |
| 73 | IR18L1082 | 300986362 | 7.01 | Long | 2.41 | 3.0 | Medium | I | 20 | I |
| 74 | IR18L1083 | 300986363 | 6.73 | Long | 2.43 | 2.8 | Medium | I | 20 | I |
| 75 | IR18L1084 | 300986364 | 6.65 | Long | 2.44 | 2.7 | Medium | HI | 20 | I |
| 76 | IR18L1085 | 300986365 | 6.87 | Long | 2.47 | 2.8 | Medium | HI/I | 20 | I |
| 77 | IR18L1086 | 300986366 | 6.88 | Long | 2.22 | 3.1 | Slender | HI | 20 | I |
| 78 | IR18L1087 | 300986367 | 6.45 | Medium | 2.47 | 2.6 | Medium | HI/I | 19.9 | L |
| 79 | IR18L1088 | 300986368 | 6.69 | Long | 2.4 | 2.8 | Medium | HI/I | 19.8 | L |
| 80 | IR18L1089 | 300986369 | 6.83 | Long | 2.28 | 3.0 | Medium | HI | 19.8 | L |
| 81 | IR18L1090 | 300986374 | 6.66 | Long | 2.42 | 2.8 | Medium | HI | 19.8 | L |
| 82 | IR18L1091 | 300986375 | 6.82 | Long | 2.26 | 3.0 | Medium | HI/I | 19.7 | L |
| 83 | IR18L1092 | 300986376 | 6.82 | Long | 2.19 | 3.1 | Slender | HI | 19.7 | L |
| 84 | IR18L1093 | 300986370 | 6.95 | Long | 2.36 | 2.9 | Medium | I | 19.6 | L |
| 85 | IR18L1094 | 300986377 | 6.56 | Medium | 2.45 | 2.7 | Medium | HI/I | 19.6 | L |
| 86 | IR18L1095 | 300986378 | 6.63 | Long | 2.43 | 2.7 | Medium | HI | 19.6 | L |
| 87 | IR18L1096 | 300986379 | 6.88 | Long | 2.12 | 3.2 | Slender | HI | 19.5 | L |
| 88 | IR18L1097 | 300986380 | 6.88 | Long | 2.17 | 3.2 | Slender | HI | 19.3 | L |
| 89 | IR18L1098 | 300986381 | 6.99 | Long | 2.25 | 3.4 | Slender | HI | 19.3 | L |
| 90 | IR18L1099 | 300986382 | 7.02 | Long | 2.23 | 3.1 | Slender | HI | 19.2 | L |
| 91 | IR18L1100 | 300986383 | 6.89 | Long | 2.29 | 3.0 | Medium | HI | 19.2 | L |
| 92 | IR18L1101 | 300986384 | 6.67 | Long | 2.25 | 3.0 | Medium | HI | 19 | L |
| 93 | IR18L1102 | 300986385 | 6.53 | Medium | 2.27 | 2.9 | Medium | HI | 18.9 | L |
| 94 | IR18L1103 | 300986386 | 6.70 | Long | 2.29 | 2.9 | Medium | HI | 18.9 | L |
| 95 | IR18L1104 | 300986372 | 7.03 | Long | 2.16 | 3.3 | Slender | HI/I | 18.8 | L |
| 96 | IR 91648-B-1-B-3-1 | 3853386 | 6.97 | Long | 2.1 | 3.3 | Slender | HI | 21.3 | I |
| 97 | MTU1010 | 1356053 | 6.61 | Long | 2.17 | 3.0 | Medium | HI/I | 20 | I |
| 99 | IR 64 | 300162714 | 6.95 | Long | 2.1 | 3.3 | Slender | I | 19.7 | L |
| 100 | IR 74371-70-1-1 | 1161409 | 6.24 | Medium | 2.33 | 2.7 | Medium | HI | 21.8 | I |
|  | **Mean** |  | **6.7** | **Long** | **2.3** | **3.1** |  | **L** | **21.5** | **I** |

Note: I-Intermediate, HI-High, L-Low
