# Supplementary material for: Genomics‐assisted breeding for successful development of multiple‐stress‐tolerant, climate‐smart rice for southern and southeastern Asia
Source: Plant Genome. 2021 Jan 12;14(1):e20074. doi: 10.1002/tpg2.20074 (PMC12806987; doi:10.1002/tpg2.20074)
Supplement: Supplementary file 6 [file TPG2-14-e20074-s006.docx]

**Table S3** Grain quality data of improved lines for product profiling to deploy in targeted breeding

| **Sl** | **Designation** | **GID** | **Physical traits by seed count (Chalkiness, grain length, grain breadth)** | | | | | | **L-B Ratio** | **Cooking traits** | | | **% Head rice** |
| --- | --- | --- | --- | --- | --- | --- | --- | --- | --- | --- | --- | --- | --- |
|  |  |  | **Grains**  **(Nos.)** | **Chalkiness** | **Grain length** | **Std. dev. (length)** | **Grain width** | **Std. dev. (width)** |  | **% Amylose** | **Amylose category** | **Gel. temp.** |  |
| 1 | IR18L1010 | 300986335 | 302 | 4.9 | 7.02 | 0.38 | 2.30 | 0.11 | 3.05 | 26.4 | H | I | 57.6 |
| 2 | IR18L1011 | 300986292 | 295 | 2.6 | 6.95 | 0.36 | 2.30 | 0.10 | 3.02 | 25.6 | H | I | 57.0 |
| 3 | IR18L1012 | 300986293 | 294 | 2.0 | 7.00 | 0.36 | 2.29 | 0.12 | 3.06 | 25.1 | H | I | 55.0 |
| 4 | IR18L1013 | 300986294 | 349 | 1.3 | 7.10 | 0.35 | 2.33 | 0.09 | 3.05 | 25.1 | H | I | 51.4 |
| 5 | IR18L1014 | 300986295 | 381 | 0.8 | 6.36 | 0.25 | 2.42 | 0.10 | 2.63 | 24.8 | I | I | 63.1 |
| 6 | IR18L1015 | 300986296 | 341 | 1.3 | 6.32 | 0.27 | 2.43 | 0.11 | 2.60 | 24.8 | I | I | 66.8 |
| 7 | IR18L1016 | 300986297 | 211 | 4.2 | 6.33 | 0.30 | 2.36 | 0.10 | 2.68 | 24.7 | I | I | 57.7 |
| 8 | IR18L1017 | 300986298 | 351 | 5.5 | 6.35 | 0.27 | 2.37 | 0.10 | 2.68 | 24.7 | I | I | 57.7 |
| 9 | IR18L1018 | 300986299 | 378 | 5.6 | 6.22 | 0.30 | 2.38 | 0.11 | 2.61 | 24.6 | I | I/L | 57.8 |
| 10 | IR18L1019 | 300986300 | 292 | 4.2 | 6.62 | 0.41 | 2.20 | 0.10 | 3.10 | 24.6 | I | I | 51.2 |
| 11 | IR18L1020 | 300986301 | 336 | 4.5 | 6.73 | 0.41 | 2.03 | 0.11 | 3.32 | 24.5 | I | I | 52.0 |
| 12 | IR18L1021 | 300986302 | 288 | 4.3 | 6.59 | 0.45 | 2.19 | 0.11 | 3.10 | 24.4 | I | I | 49.8 |
| 13 | IR18L1022 | 300986303 | 349 | 4.3 | 6.67 | 0.40 | 2.17 | 0.11 | 3.07 | 24.3 | I | I | 52.3 |
| 14 | IR18L1023 | 300986304 | 314 | 6.9 | 6.74 | 0.44 | 2.21 | 0.12 | 3.05 | 24.0 | I | I | 52.7 |
| 15 | IR18L1024 | 300986305 | 390 | 5.3 | 6.16 | 0.27 | 2.43 | 0.11 | 2.53 | 24.0 | I | I | 62.2 |
| 16 | IR18L1025 | 300986306 | 281 | 5.7 | 6.25 | 0.28 | 2.36 | 0.10 | 2.65 | 23.7 | I | I | 58.5 |
| 17 | IR18L1026 | 300986314 | 308 | 4.1 | 6.24 | 0.27 | 2.38 | 0.12 | 2.62 | 23.7 | I | I | 65.8 |
| 18 | IR18L1027 | 300986307 | 316 | 5.9 | 6.76 | 0.46 | 2.23 | 0.12 | 3.03 | 23.7 | I | I | 47.2 |
| 19 | IR18L1028 | 300986315 | 317 | 4.2 | 6.79 | 0.42 | 2.26 | 0.10 | 3.10 | 23.6 | I | I | 51.8 |
| 20 | IR18L1029 | 300986316 | 288 | 6.1 | 6.83 | 0.42 | 2.24 | 0.12 | 3.05 | 23.5 | I | I | 49.6 |
| 21 | IR18L1030 | 300986317 | 332 | 3.5 | 6.84 | 0.40 | 2.26 | 0.11 | 3.03 | 23.4 | I | I | 57.0 |
| 22 | IR18L1031 | 300986308 | 319 | 3.4 | 6.82 | 0.39 | 2.20 | 0.10 | 3.10 | 23.4 | I | I | 58.6 |
| 23 | IR18L1032 | 300986318 | 330 | 5.4 | 6.84 | 0.40 | 2.20 | 0.11 | 3.11 | 23.0 | I | I | 51.5 |
| 24 | IR18L1033 | 300986319 | 360 | 4.8 | 6.78 | 0.35 | 2.25 | 0.11 | 3.01 | 22.9 | I | I | 53.4 |
| 25 | IR18L1034 | 300986320 | 405 | 1.3 | 6.88 | 0.38 | 2.20 | 0.10 | 3.13 | 22.9 | I | HI | 59.0 |
| 26 | IR18L1035 | 300986309 | 341 | 3.7 | 6.75 | 0.39 | 2.19 | 0.11 | 3.08 | 22.8 | I | I | 57.2 |
| 27 | IR18L1036 | 300986322 | 338 | 5.3 | 6.65 | 0.39 | 2.15 | 0.12 | 3.09 | 22.6 | I | I | 52.2 |
| 28 | IR18L1037 | 300986323 | 320 | 2.6 | 6.63 | 0.33 | 2.17 | 0.11 | 3.36 | 22.3 | I | I | 54.8 |
| 29 | IR18L1038 | 300986324 | 322 | 2.2 | 6.82 | 0.37 | 2.23 | 0.10 | 3.06 | 22.2 | I | I | 52.6 |
| 30 | IR18L1039 | 300986310 | 379 | 5.1 | 6.88 | 0.35 | 2.32 | 0.11 | 2.97 | 22.2 | I | I | 50.6 |
| 31 | IR18L1040 | 300986325 | 312 | 4.5 | 6.80 | 0.33 | 2.30 | 0.11 | 2.96 | 22.2 | I | I | 52.5 |
| 32 | IR18L1041 | 300986326 | 300 | 2.7 | 6.72 | 0.37 | 2.18 | 0.11 | 3.08 | 22.2 | I | HI/I | 46.1 |
| 33 | IR18L1042 | 300986327 | 306 | 1.9 | 6.95 | 0.34 | 2.15 | 0.11 | 3.23 | 22.2 | I | I | 61.3 |
| 34 | IR18L1043 | 300986311 | 287 | 1.8 | 6.94 | 0.31 | 2.13 | 0.12 | 3.26 | 22.1 | I | I | 65.4 |
| 35 | IR18L1044 | 300986328 | 306 | 3.5 | 6.90 | 0.36 | 2.29 | 0.11 | 3.01 | 21.9 | I | HI | 61.2 |
| 36 | IR18L1045 | 300986329 | 358 | 1.2 | 6.89 | 0.35 | 2.30 | 0.11 | 3.10 | 21.9 | I | I | 64.1 |
| 37 | IR18L1046 | 300986336 | 344 | 7.9 | 6.46 | 0.30 | 2.40 | 0.11 | 2.69 | 21.8 | I | L | 47.2 |
| 38 | IR18L1047 | 300986330 | 310 | 2.3 | 6.94 | 0.37 | 2.02 | 0.11 | 3.44 | 21.7 | I | HI/I | 59.1 |
| 39 | IR18L1048 | 300986331 | 311 | 3.0 | 6.81 | 0.41 | 2.35 | 0.11 | 3.00 | 21.4 | I | I | 55.0 |
| 40 | IR18L1049 | 300986332 | 326 | 22.8 | 6.52 | 0.31 | 2.25 | 0.11 | 2.90 | 21.4 | I | I | 52.6 |
| 41 | IR18L1050 | 300986312 | 356 | 5.0 | 6.51 | 0.28 | 2.44 | 0.12 | 2.67 | 21.4 | I | I | 56.9 |
| 42 | IR18L1051 | 300986333 | 346 | 5.8 | 6.38 | 0.26 | 2.48 | 0.12 | 2.57 | 21.3 | I | I | 55.2 |
| 43 | IR18L1052 | 300986334 | 327 | 5.5 | 6.47 | 0.30 | 2.46 | 0.12 | 2.63 | 21.3 | I | I | 61.0 |
| 44 | IR18L1053 | 300986321 | 318 | 2.7 | 6.76 | 0.37 | 2.21 | 0.10 | 3.16 | 21.3 | I | HI | 49.8 |
| 45 | IR18L1054 | 300986339 | 297 | 15.5 | 6.77 | 0.36 | 2.21 | 0.10 | 3.46 | 21.3 | I | HI | 31.4 |
| 46 | IR18L1055 | 300986340 | 313 | 3.7 | 7.03 | 0.47 | 2.15 | 0.11 | 3.27 | 21.2 | I | I | 44.1 |
| 47 | IR18L1056 | 300986341 | 311 | 1.9 | 7.04 | 0.43 | 2.12 | 0.12 | 3.32 | 21.2 | I | HI | 51.1 |
| 48 | IR18L1057 | 300986338 | 310 | 2.7 | 7.05 | 0.42 | 2.17 | 0.11 | 3.25 | 21.1 | I | HI | 47.9 |
| 49 | IR18L1058 | 300986342 | 291 | 6.5 | 6.83 | 0.34 | 2.47 | 0.12 | 2.77 | 21.0 | I | HI | 53.3 |
| 50 | IR18L1059 | 300986343 | 325 | 2.7 | 6.72 | 0.35 | 2.37 | 0.13 | 2.84 | 20.9 | I | HI/I | 63.0 |
| 51 | IR18L1060 | 300986344 | 286 | 2.4 | 6.80 | 0.33 | 2.45 | 0.12 | 2.78 | 20.7 | I | I | 60.3 |
| 52 | IR18L1061 | 300986337 | 317 | 2.8 | 6.73 | 0.39 | 2.44 | 0.13 | 2.76 | 20.7 | I | I | 59.9 |
| 53 | IR18L1062 | 300986345 | 274 | 2.8 | 6.73 | 0.30 | 2.03 | 0.11 | 3.34 | 20.6 | I | HI/I | 57.0 |
| 54 | IR18L1063 | 300986346 | 285 | 2.7 | 6.87 | 0.41 | 2.10 | 0.12 | 3.27 | 20.6 | I | HI | 51.1 |
| 55 | IR18L1064 | 300986347 | 264 | 5.7 | 6.81 | 0.45 | 2.22 | 0.12 | 3.07 | 20.6 | I | HI/I | 46.3 |
| 56 | IR18L1065 | 300986313 | 339 | 4.0 | 6.53 | 0.36 | 2.17 | 0.11 | 3.10 | 20.6 | I | HI/I | 57.7 |
| 57 | IR18L1066 | 300986348 | 313 | 5.5 | 6.57 | 0.38 | 2.16 | 0.11 | 3.04 | 20.6 | I | HI/I | 57.7 |
| 58 | IR18L1067 | 300986349 | 292 | 2.0 | 7.00 | 0.49 | 2.14 | 0.12 | 3.27 | 20.5 | I | HI/I | 46.9 |
| 59 | IR18L1068 | 300986371 | 374 | 1.7 | 6.86 | 0.43 | 2.10 | 0.11 | 3.27 | 20.5 | I | HI/I | 53.1 |
| 60 | IR18L1069 | 300986350 | 319 | 0.8 | 7.06 | 0.40 | 2.31 | 0.11 | 3.06 | 20.5 | I | HI | 61.9 |
| 61 | IR18L1070 | 300986351 | 280 | 0.7 | 7.19 | 0.42 | 2.35 | 0.12 | 3.06 | 20.4 | I | I | 63.7 |
| 62 | IR18L1071 | 300986352 | 347 | 1.9 | 6.76 | 0.44 | 2.07 | 0.12 | 3.27 | 20.4 | I | I | 54.3 |
| 63 | IR18L1072 | 300986353 | 304 | 1.7 | 6.75 | 0.47 | 2.24 | 0.11 | 3.01 | 20.4 | I | HI | 53.6 |
| 64 | IR18L1073 | 300986354 | 315 | 5.2 | 6.78 | 0.33 | 2.41 | 0.12 | 2.81 | 20.4 | I | HI | 58.6 |
| 65 | IR18L1074 | 300986355 | 349 | 5.3 | 6.69 | 0.33 | 2.40 | 0.11 | 2.79 | 20.3 | I | I | 53.0 |
| 66 | IR18L1075 | 300986373 | 324 | 3.0 | 6.71 | 0.32 | 2.41 | 0.11 | 2.78 | 20.3 | I | HI | 59.4 |
| 67 | IR18L1076 | 300986356 | 329 | 1.9 | 5.90 | 0.26 | 2.48 | 0.12 | 2.38 | 20.3 | I | HI/I | 61.3 |
| 68 | IR18L1077 | 300986357 | 328 | 1.7 | 6.14 | 0.27 | 2.47 | 0.11 | 2.49 | 20.2 | I | HI/I | 56.9 |
| 69 | IR18L1078 | 300986358 | 245 | 6.0 | 6.85 | 0.37 | 2.09 | 0.11 | 3.28 | 20.1 | I | I | 54.8 |
| 70 | IR18L1079 | 300986359 | 376 | 3.4 | 6.98 | 0.38 | 2.31 | 0.11 | 3.02 | 20.1 | I | HI/I | 52.9 |
| 71 | IR18L1080 | 300986360 | 324 | 1.8 | 6.76 | 0.45 | 2.12 | 0.10 | 3.19 | 20.1 | I | HI | 56.5 |
| 72 | IR18L1081 | 300986361 | 336 | 1.4 | 6.78 | 0.43 | 2.16 | 0.12 | 3.14 | 20.1 | I | HI/I | 57.9 |
| 73 | IR18L1082 | 300986362 | 303 | 6.4 | 7.01 | 0.41 | 2.41 | 0.13 | 2.99 | 20.0 | I | I | 54.6 |
| 74 | IR18L1083 | 300986363 | 288 | 4.9 | 6.73 | 0.32 | 2.43 | 0.11 | 2.77 | 20.0 | I | I | 61.4 |
| 75 | IR18L1084 | 300986364 | 346 | 4.2 | 6.65 | 0.32 | 2.44 | 0.13 | 2.73 | 20.0 | I | HI | 62.7 |
| 76 | IR18L1085 | 300986365 | 279 | 6.2 | 6.87 | 0.30 | 2.47 | 0.12 | 2.78 | 20.0 | I | HI/I | 61.6 |
| 77 | IR18L1086 | 300986366 | 299 | 1.7 | 6.88 | 0.43 | 2.22 | 0.10 | 3.10 | 20.0 | I | HI | 44.8 |
| 78 | IR18L1087 | 300986367 | 325 | 3.1 | 6.45 | 0.32 | 2.47 | 0.11 | 2.61 | 19.9 | L | HI/I | 60.6 |
| 79 | IR18L1088 | 300986368 | 304 | 7.9 | 6.69 | 0.37 | 2.40 | 0.11 | 2.79 | 19.8 | L | HI/I | 58.3 |
| 80 | IR18L1089 | 300986369 | 275 | 0.9 | 6.83 | 0.39 | 2.28 | 0.11 | 3.00 | 19.8 | L | HI | 61.2 |
| 81 | IR18L1090 | 300986374 | 329 | 18.6 | 6.66 | 0.28 | 2.42 | 0.12 | 2.75 | 19.8 | L | HI | 30.6 |
| 82 | IR18L1091 | 300986375 | 318 | 1.3 | 6.82 | 0.39 | 2.26 | 0.12 | 3.02 | 19.7 | L | HI/I | 57.8 |
| 83 | IR18L1092 | 300986376 | 317 | 16.6 | 6.82 | 0.39 | 2.19 | 0.13 | 3.11 | 19.7 | L | HI | 40.6 |
| 84 | IR18L1093 | 300986370 | 269 | 13.6 | 6.95 | 0.40 | 2.36 | 0.14 | 2.94 | 19.6 | L | I | 37.6 |
| 85 | IR18L1094 | 300986377 | 324 | 8.0 | 6.56 | 0.32 | 2.45 | 0.13 | 2.68 | 19.6 | L | HI/I | 42.8 |
| 86 | IR18L1095 | 300986378 | 325 | 11.0 | 6.63 | 0.29 | 2.43 | 0.14 | 2.73 | 19.6 | L | HI | 39.8 |
| 87 | IR18L1096 | 300986379 | 263 | 9.9 | 6.88 | 0.47 | 2.12 | 0.12 | 3.25 | 19.5 | L | HI | 45.9 |
| 88 | IR18L1097 | 300986380 | 290 | 16.3 | 6.88 | 0.45 | 2.17 | 0.12 | 3.17 | 19.3 | L | HI | 37.0 |
| 89 | IR18L1098 | 300986381 | 337 | 5.5 | 6.99 | 0.38 | 2.25 | 0.12 | 3.41 | 19.3 | L | HI | 46.3 |
| 90 | IR18L1099 | 300986382 | 297 | 1.8 | 7.02 | 0.42 | 2.23 | 0.13 | 3.15 | 19.2 | L | HI | 49.6 |
| 91 | IR18L1100 | 300986383 | 356 | 1.4 | 6.89 | 0.39 | 2.29 | 0.11 | 3.01 | 19.2 | L | HI | 63.3 |
| 92 | IR18L1101 | 300986384 | 258 | 5.1 | 6.67 | 0.42 | 2.25 | 0.13 | 2.96 | 19.0 | L | HI | 33.4 |
| 93 | IR18L1102 | 300986385 | 294 | 4.7 | 6.53 | 0.44 | 2.27 | 0.12 | 2.88 | 18.9 | L | HI | 48.5 |
| 94 | IR18L1103 | 300986386 | 277 | 0.4 | 6.70 | 0.39 | 2.29 | 0.12 | 2.93 | 18.9 | L | HI | 64.4 |
| 95 | IR18L1104 | 300986372 | 293 | 6.0 | 7.03 | 0.38 | 2.16 | 0.14 | 3.25 | 18.8 | L | HI/I | 38.1 |
| 96 | IR 91648-B-1-B-3-1 | 3853386 | 313 | 1.55 | 6.97 | 0.41 | 2.1 | 0.12 | 3.31 | 21.3 | I | HI | 59 |
| 97 | MTU1010 | 1356053 | 336 | 4.79 | 6.61 | 0.37 | 2.17 | 0.12 | 3.04 | 20 | I | HI/I | 53 |
| 98 | Swarna | 4026120 | 221 | 0.78 | 5.42 | 0.27 | 2.37 | 0.12 | 2.28 | 25.2 | H | I | 64.5 |
| 99 | IR 64 | 300162714 | 234 | 9.37 | 6.95 | 0.47 | 2.1 | 0.14 | 3.3 | 19.7 | L | HI | 45.8 |
| 100 | IR 74371-70-1-1 | 1161409 | 317 | 5.32 | 6.24 | 0.29 | 2.33 | 0.13 | 2.67 | 21.8 | I | L | 51.8 |
|  | **Mean** |  | **314** | **4.7** | **6.7** | **0.4** | **2.3** | **0.1** | **3.1** | **21.5** | **I** | **I** | **53.9** |

Note: I-Intermediate, HI-High, L-Low
