# Supplementary material for: Genomics‐assisted breeding for successful development of multiple‐stress‐tolerant, climate‐smart rice for southern and southeastern Asia
Source: Plant Genome. 2021 Jan 12;14(1):e20074. doi: 10.1002/tpg2.20074 (PMC12806987; doi:10.1002/tpg2.20074)
Supplement: Supplementary file 7 [file TPG2-14-e20074-s007.docx]

**Table S2** Gall midge resistant improved lines

| **Sl** | **Designation** | **Tiller (Nos.)** | **% Onion Shoot** | **Reaction** |
| --- | --- | --- | --- | --- |
| 1 | IR18L1049 | 19 | 0.00 | HR |
| 2 | IR18L1056 | 33 | 0.00 | HR |
| 3 | IR18L1127 | 23 | 0.00 | HR |
| 4 | IR18L1157 | 34 | 18.38 | MS |
| 5 | IR18L1147 | 23 | 0.00 | HR |
| 6 | IR18L1137 | 40 | 0.00 | HR |
| 7 | IR18L1142 | 29 | 2.22 | R |
| 8 | IR18L1130 | 28 | 0.00 | HR |
| 9 | IR18L1144 | 25 | 0.00 | HR |
| 10 | BRRI dhan33(R. Ck) | 42 | 0.00 | HR |
| 11 | BRRI Dhan 49(S. Ck) | 70 | 91.00 | S |
| 12 | BRRI dhan33(R. Ck) | 40 | 0.00 | HR |
| 13 | BRRI dhan49 (S.Ck) | 58 | 88.80 | S |
| 14 | BRRI dhan33 (R. Ck) | 38 | 0.00 | HR |
| 15 | BRRI dhan49 (S.Ck) | 59 | 89.74 | S |

Note: MS: Moderately Susceptible, MR: Moderately Resistant, S: Susceptible R: Resistant, R.Ck: Resistant Check, S.Ck.: Susceptible Check
